# Enteric Neurons and Systemic Signals Couple Nutritional and Reproductive Status with Intestinal Homeostasis

**DOI:** 10.1016/j.cmet.2010.12.010

**Published:** 2011-01-05

**Authors:** Paola Cognigni, Andrew P. Bailey, Irene Miguel-Aliaga

**Affiliations:** 1 Department of Zoology, University of Cambridge, Downing Street, Cambridge CB2 3EJ, UK; 2 Division of Developmental Neurobiology, MRC National Institute for Medical Research, The Ridgeway, Mill Hill, London NW7 1AA, UK

## Abstract

The gastrointestinal tract is emerging as a key regulator of appetite and metabolism, but daunting neuroanatomical complexity has hampered identification of the relevant signals. Invertebrate models could provide a simple and genetically amenable alternative, but their autonomic nervous system and its visceral functions remain largely unexplored. Here we develop a quantitative method based on defecation behavior to uncover a central role for the *Drosophila* intestine in the regulation of nutrient intake, fluid, and ion balance. We then identify a key homeostatic role for autonomic neurons and hormones, including a brain-gut circuit of insulin-producing neurons modulating appetite, a vasopressin-like system essential for fluid homeostasis, and enteric neurons mediating sex peptide-induced changes in intestinal physiology. These conserved mechanisms of visceral control, analogous to those found in the enteric nervous system and hypothalamic/pituitary axis, enable the study of autonomic control in a model organism that has proved instrumental in understanding sensory and motor systems.

## Graphical abstract

**Graphical Abstract:**

**Figure:**
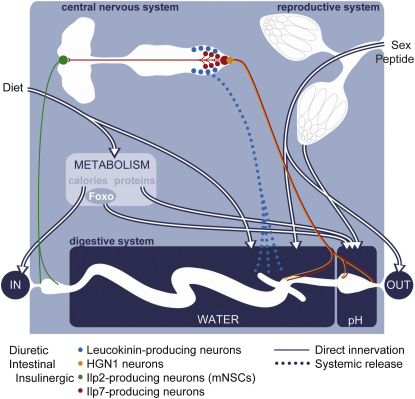

**Highlights:**

► A metabolic behavior revealing a key homeostatic role for the invertebrate intestine ► Enteric neurons couple reproductive state with intestinal physiology ► An insulinergic brain-gut neuronal circuit adjusts feeding to nutritional conditions ► A vasopressin-like system of central neurons is essential for water homeostasis

## Introduction

The homeostatic requirements arising from multicellularity contributed to the evolution of complex nervous systems able to adjust both behavior and visceral function on the basis of information about metabolic status and nutrient availability (Iversen et al., 2000). In mammals, the sensors and effectors of homeostatic metabolic changes are specific neuronal populations and brain/gut hormones which, when deregulated, can contribute to increasingly prevalent medical conditions such as diabetes and metabolic syndrome (Sandoval et al., 2008; Strader and Woods, 2005). In this regard, the gastrointestinal tract is emerging as an important source of both neural and systemic signals regulating appetite and body weight (Sandoval et al., 2008; Strader and Woods, 2005). However, the intricate nature of these signals can make their study a challenging task. Indeed, autonomic fibers assemble into structurally complex and diverse neural circuits with the enteric nervous system, which, in humans, consists of ca. 500 million neurons. Moreover, the crosstalk between the digestive and nervous systems can also be mediated by a suite of enteroendocrine hormones, as well as those produced centrally by the hypothalamic-pituitary-adrenal axis (Furness, 2006; Nussey and Whitehead, 2001). Such regulatory complexity is not conducive to the genetic study of homeostasis, which would benefit from manipulation of gene expression or neuronal function with temporal and spatial resolution.

The simpler neuroanatomy and genetic amenability of invertebrate systems could provide an entry point into the study of metabolic homeostasis in more complex organisms. Recent studies have established functional orthology between mammalian and insect hormones, thereby contributing to the emergence of *Drosophila* as a model for the study of metabolism (Buch and Pankratz, 2009; Leopold and Perrimon, 2007). However, progress has been hampered by both our lack of detailed knowledge of the fly's autonomic neural circuits and the limited repertoire of behavioral and physiological readouts with which to investigate the internal control of nutrient intake and utilization. Consequently, the homeostatic regulation of visceral functions subsequent to the decision of whether or not to eat remains largely unexplored.

Here we investigate the nature and relevance of visceral homeostasis in *Drosophila* by developing an integrative method to quantify metabolic features, which we combine with mutant analyses and the selective manipulation of neuronal subsets. This uncovers a central role for the invertebrate intestine in the integration of neural, nutritional, and reproductive information to adjust nutrient intake and utilization. Furthermore, it reveals extensive and essential autonomic and systemic control of visceral functions by both novel and conserved neural substrates, analogous to those found in the mammalian enteric nervous system and hypothalamic/pituitary gland.

## Results

### Distinct Domains of the *Drosophila* Intestine Are Innervated by Both Efferent and Sensory Neurons

**Figure 1:**
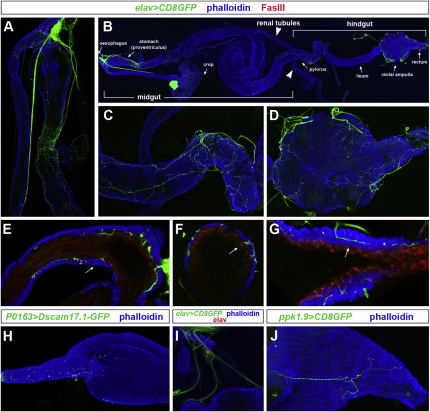
Innervation of the Adult Intestine Smooth muscles are visualized with phalloidin (in blue). Anterior (oral) is to the left in all images except for (A) and (E) (where oral is toward the top) and (I). (A) Anterior midgut. Note the nerve fibers emanating from the corpus cardiacum/hypocerebral ganglion. (B) Whole digestive tract, showing three innervated portions. The anterior-most segment comprises the esophagus, esophageal valve, and anterior midgut (proventriculus, analogous to the human stomach, Wigglesworth, 1972) and is innervated both centrally and by the peripheral corpus cardiacum/hypocerebral ganglion. Following a midgut segment devoid of innervations, central fibers innervate the pyloric valve (which regulates the entry of midgut and renal tubule contents into the hindgut) and, following a short noninnervated ileum segment, the terminal portion of the intestine including the rectal valve, rectal ampulla, and rectum. This innervation pattern was confirmed with the panneuronal *n-syb-Gal4* line (Figure S1) and eight other broadly expressed driver lines or antibodies (data not shown). (C and D) Innervation of the midgut/hindgut junction (pylorus, C) and the rectal ampulla (D). (E–G) Neurites projecting through contiguous circular smooth muscles (arrows) toward the epithelium (stained with anti-FasIII) of the crop duct (E) and the hindgut (F), and the underlying layer of longitudinal muscles in the rectum (G). (H) Sensory neurites (as visualized by expression of the dendritic marker *Dscam17.1-GFP* expressed from the peripheral neuron driver *P0163-Gal4*) in the esophagus. (I) A peripheral neuron innervating the rectal ampulla, as revealed by its expression of elav (antibody staining, red nucleus) and a membrane-tagged GFP expressed from *elav-Gal4*. (J) Sensory innervation of the rectal ampulla, as revealed by the *ppk1.9-Gal4* reporter. See also Figure S1.

In contrast to the sensory and motor systems, the visceral nervous system of *Drosophila* was largely uncharacterized. The use of multiple markers and reporters revealed complex innervation of the adult intestine, which is confined to three distinct portions (Figures 1A–1D and see Figures S1A–S1C available online). The presence of neurites positive for the motor neuron reporter *OK371-Gal4* (Figures S1D–S1F) in these three innervated segments containing valves or sphincters suggests modulation of intestinal transit by neural control of smooth muscle function. Interestingly, in addition to this superficial innervation of intestinal muscles, we also observed a subset of fibers which, analogous to mammalian submucosal fibers (Furness, 2006), project through contiguous circular muscles toward the underlying epithelial layer (Figures 1E–1G).

While at least some of the enteric fibers innervating the anterior portion of the digestive tract originate from peripheral cell bodies (Figure 1A and Figure S1A), the other two innervated portions (pylorus and rectal ampulla/rectum) receive innervation from cell bodies located in the central nervous system (Figures S1J and S1K). However, not all of the innervation of the digestive tract is efferent. Indeed, the combined use of sensory neuron drivers and dendritic markers revealed sensory innervation of the esophagus and proventriculus and the presence of one to three peripheral sensory neurons innervating the posterior portion of the hindgut (Figures 1H–1J and Figures S1G–S1I).

In sum, our anatomical analyses reveal extensive sensory and efferent innervation of both intestinal muscles and epithelial cells, which is confined to discrete portions of the digestive tract.

### A New Metabolic Behavior Reveals Homeostatic Control of Food Intake, Intestinal Transit, Diuresis, and Acid-Base Balance in Adult Flies

**Figure 2:**
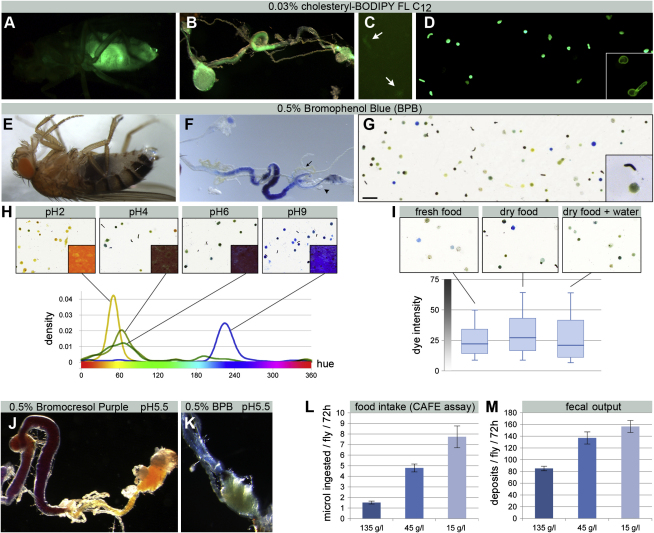
New Quantitative Methods to Assess Intestinal Functions (A–D) Strong fluorescence is apparent in intact (A) and dissected guts (B) and the excreta of Fluoropoo-fed flies, both on food (C, arrows) and on the clear walls of vials (D). The deposits from a mixed population have two distinct shapes (D, inset): round and oblong. (E–G) Shown are intestinal contents in BPB-fed intact flies (E) and dissected intestinal tracts (F). Like with Fluoropoo, no dye is apparent in the renal tubules (F, arrow) or fat body (F, arrowhead). (G) BPB-labeled deposits differ in their color and concentration. Round and oblong shapes are also apparent (G, inset). Oblong deposits are typically more concentrated. Scale bar, 1 mm. (H) Defined amounts of acid or base change the pH and color of BPB-supplemented food (top panels, insets). The color range of the resulting deposits shifts accordingly (top panels). Hue density distributions indicate that the majority of deposits cluster at specific hue points depending on the pH of the food. (I) Male flies fed drier food (standard food dried for 2 hr at 40°C) produce more concentrated deposits (increased average dye intensity, p < 0.0001, Mann-Whitney U test, n = 10 flies/set). This phenotype is rescued when flies fed on this dry food have additional access to water (p = 0.015 when compared to dry food, not significant when compared to standard food, Mann-Whitney U test, n = 10 flies/set). (J and K) Two pH indicators with a different pH range (see the Experimental Procedures for details) reveal pH changes along the intestinal tract under normal conditions (standard diet, pH 5.5). Hindgut contents are acidified just posterior to the renal tubules (J, purple to light orange). Further acidification occurs just prior to excretion in the rectum (K, blue to yellow). (L) Compensatory increase in feeding upon food dilution in the 135–15 g/L range, as measured by the CAFE assay (p < 0.001 135 g/L versus 45 g/L, p = 0.0095 45 g/L versus 15 g/L, p = 0.0018 135 g/L versus 15 g/L, Mann-Whitney U test, n = 14 flies/set, although only five flies were left in the 15 g/L group after 72 hr due to the high lethality caused by this low-calorie diet). (M) Differences in fecal output resulting from the same dilution series quantified using our assay (p < 0.001 135 g/L versus 45 g/L, p = 0.19 45 g/L versus 15 g/L, p < 0.0001 135 g/L versus 15 g/L, Mann-Whitney U test, n = 15 flies/set). No lethality was observed by 72 hr in the low-calorie 15 g/L diet. See also Figure S2, Table S1, and the Experimental Procedures for technical details and quantification protocols. Graphs show average ± standard error of the mean.

A functional readout was required to explore the homeostatic significance of this innervation. We reasoned that the material excreted by adult flies may provide an integrated readout of digestive and excretory status since the renal tubules discharge into the intestine at the midgut-hindgut boundary (Figure 1B). To measure overall defecation rates, we supplemented standard fly food with a modified lipid probe which only becomes fluorescent after exposure to the fly's digestive tract (referred to as Fluoropoo, Figures 2A–2D, see the Experimental Procedures for details), thereby allowing us to quantify the total number of excreta produced (including those deposited on the food, Figure 2C). We found defecation rate to be modulated by diet in a manner predictive of food intake, as measured by the CAFE assay (Ja et al., 2007) (Figures 2L and 2M). Although this is a less direct measure of food intake than the CAFE assay and requires two additional controls (Table S1), it can be performed in dietary conditions more comparable to those in which the flies were bred.

In addition to providing quantitative information about intestinal emptying rate, we were able to extract further information about the nature of *Drosophila* excreta by using the pH indicator dye Bromophenol blue (BPB, Figures 2E–2G) to obtain colored deposits, which we then analyzed semiautomatedly to extract quantitative information about size, shape, concentration, and color (Figure S2, Table S1, and Experimental Procedures for details). The use of another pH indicator and experiments with diets of defined acidity and water content validated the use of this method to quantify the pH and fluid content of excreta, and revealed dietary modulation of both (Figures 2H–2K).

In sum, this semiautomatic analysis of fly excreta provides an integrated physiological readout for food intake, acid-base balance and diuresis, which reveals dietary modulation of intestinal emptying rate, pH, and fluid content.

### Intestinal Acid-Base Homeostasis Is Modulated by Diet and Internal Reproductive State

**Figure 3:**
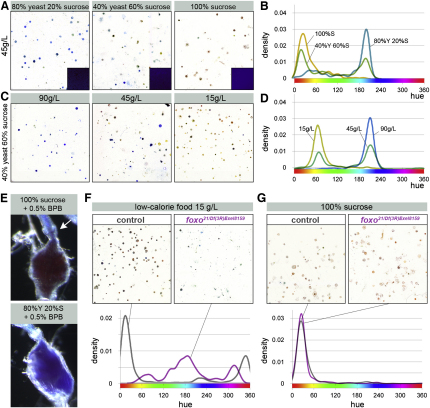
Dietary Regulation of Intestinal Acid-Base Balance (A) Regulation of fecal pH by diet composition. pH changes result from differential metabolism because all three diets were adjusted to the same initial pH (food color is shown in insets). The total concentration of all three diets was 45 g/L. (B) Hue density plots of excreta resulting from 100% sucrose (yellow plot), 40% yeast/60% sucrose (green), and 80% yeast/20% sucrose (blue) diets (p < 0.0001 for all three comparisons, two-sample Kolmogorov-Smirnov test). (C) Regulation of fecal pH by progressive dilution of a balanced diet with a constant 40% yeast, 60% sucrose ratio. (D) Hue density plots of excreta resulting from 90 g/L (blue), 45 g/L (green), and 15 g/L (yellow) diets (p < 0.0001 for all three comparisons, two-sample Kolmogorov-Smirnov test). (E) The acidification of excreta in sugar-fed flies occurs in the rectum. Note the change from blue to orange/red immediately posterior to the rectal valve (arrow). By contrast, intestinal contents remain basic in the hindgut of flies that have been fed a high fat/protein diet. (F) In *foxo* mutants, the shift to a more acidic pH triggered by a low-calorie diet is partly impaired, unlike that of control (*w^1118^*) flies. The hue density plot of *foxo* mutants does not peak at the red/orange hues indicative of acidic excreta (p < 0.0001, two-sample Kolmogorov-Smirnov test). The pH of their excreta and intestinal morphology was indistinguishable from that of control flies in fully fed conditions (Figures S3B–S3I and data not shown). In the next 48 hr, *foxo* mutants do shift to a more acidic pH fully, but this is accompanied by very high lethality (data not shown). The same results were obtained with a different *foxo* mutant combination (*foxo^w24^/foxo^21^*, data not shown). (G) The effect of a sugar-only diet on acid-base balance is not affected in *foxo* mutants. The hue density plots of both controls and *foxo* mutants peak at red/orange hues. See also Figure S3.

The dietary modulation of excreta pointed to a homeostatic role for the intestine. Experiments with defined diets containing different sucrose/protein ratios, but which were otherwise the same pH (Figure 3A, insets), revealed that the posterior hindgut (analogous to the large intestine) differentially adjusts the final pH of excreta when flies are fed on diets known to lead to distinct metabolic states (Zinke et al., 2002 and Figure S3A): indeed, acidification of deposits and hindgut contents was always apparent in sucrose-fed flies but was never observed in a protein-rich diet (Figures 3A, 3B, and 3E). Interestingly, dietary restriction (a regime that can lead to life span extension in many organisms and which, in *Drosophila*, is implemented by dilution of the food medium in water [Piper et al., 2005]) also led to persistent acidification of intestinal contents (Figures 3C and 3D). This effect, observed under conditions which did not affect the dietary sugar/yeast ratio, suggested that the dietary modulation of acid-base balance does not passively result from the differential utilization of specific metabolic pathways. Analysis of mutants for the transcription factor *foxo*, which orchestrates changes in metabolic gene expression in response to starvation (Teleman et al., 2008), revealed that while the pH shift induced by a sugar-only diet occurred normally in these mutants (Figure 3G), their shift to a more acidic pH in response to nutrient scarcity was impaired (Figure 3F). This confirmed that the *Drosophila* intestine modulates the final composition of intestinal contents in response to diet-induced changes in internal metabolism, and indicated that diet composition and nutrient concentration impact on intestinal pH through two distinct mechanisms.

**Figure 4:**
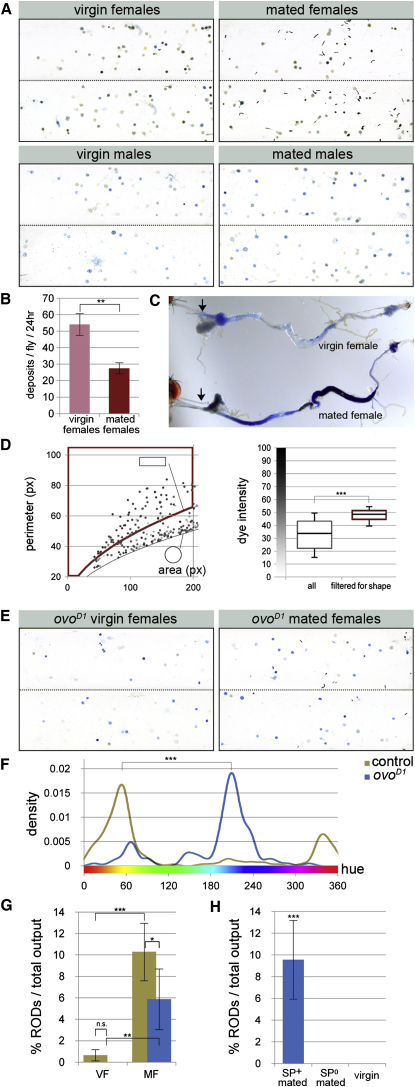
Effects of Reproduction on Intestinal Physiology (A) Shown is representative fecal output of virgin or mated males and females. Although each subpanel consists of excreta of three to six flies, quantitative analyses were conducted on larger groups of flies (n = 9–12 flies, see Figure S4 for details). (B) The defecation rate of wild-type mated females is lower than that of virgin females (p = 0.007, Mann-Whitney U test, n = 10 flies/set). (C) The intestinal contents of mated females are more concentrated than those of virgin females. Concentration is already apparent in the crop and anterior midgut (arrows), suggesting a change in intestinal fluid retention. (D) Oblong deposits are very concentrated excreta. Left graph shows the distribution of deposits by area and perimeter in relation to circular shapes (lower black line, p = 2π√(a/π)) and an arbitrarily defined oblong shape (a 3 × 1 rectangle, upper black line, p = 8√[a/3]); dye intensity is represented in grayscale for each deposit. The subpopulation of noncircular deposits (p ≥ 8√[a/3] and area ≤ 200 px, boxed in red) is significantly darker than the population as a whole (right graph, p < 0.0001, Mann-Whitney U test, n = 12 flies). (E) Shown are fecal outputs of *ovo^D1^* virgin and mated females, which lack functional ovaries. (F) The majority of excreta in *ovo^D1^* virgin females are blue (basic). This contrasts with the wild-type profile of control OreR virgin females, which peaks around more acidic orange/green hues (p < 0.0001, two-sample Kolmogorov-Smirnov test). (G) Mating increases the relative frequency of concentrated RODs in both *w^1118^* controls (brown bars) and *ovo^D1^* females (blue bars, p < 0.001 and p = 0.009, respectively, Mann-Whitney U test, n = 9–10 flies/set). VF, virgin females; MF, mated females. (H) *ovo^D1^* females do not produce RODs when they are mated to sex peptide null (*SP^0^/SP^Δ130^*) males (p < 0.001 when compared to *ovo^D1^* females mated to control *SP^+^/SP^Δ130^* males, Mann-Whitney U test and Fisher's combined probability test, n = 10–13 flies/set, two different experiments). See also Figures S4 and S5. Graphs show average ± standard error of the mean.

We then wondered whether, in addition to environmental dietary challenges, internal nutritional challenges such as those associated with reproduction also impact on intestinal acid-base homeostasis. The sexually dimorphic nature of fecal output (Figure 4A, Figures S4A and S4B) is consistent with this hypothesis: females, which produce large and nutrient-rich gametes, show acidification of excreta reminiscent of that caused by the lack of nutrients or protein. Modulation of acid-base homeostasis by egg production was corroborated by the fecal output of *ovo^D1^* sterile females, which is more similar to that of wild-type virgin males than to that of wild-type virgin females (Figures 4A, 4E, and 4F, and Figure S5A), and was further confirmed by additional genetic manipulations which differentially interfered with oogenesis and/or egg laying (Figure S5A). These genetic manipulations also suggested that the differential pH is specifically caused by the nutritional demands associated with vitellogenesis.

Together, these results indicate that, like its human counterpart (Charney and Dagher, 1996), the *Drosophila* large intestine modulates the final composition of intestinal contents. Interestingly, it can do so in response to changes in internal metabolism, a role that has been proposed but not directly shown for human colonic absorption (Charney and Dagher, 1996).

### A Very Small Subset of Intestinal Neurons Modulates Intestinal Fluid Balance in Response to a Reproductive Hormone

The complex modulation of enteric physiology during pregnancy in humans (Keller et al., 2008) prompted us to investigate whether, in addition to pH, reproduction affected additional aspects of intestinal homeostasis. Our assay revealed dramatic changes triggered in females by mating. First, mated females defecate on food more often than virgin females or males do: probably a result of their increased egg laying activity (Figure S4C). Second, in spite of increasing their food intake (Carvalho et al., 2006), their intestinal transit is markedly decreased (Figure 4B). This is accompanied by a remarkable change in intestinal physiology, whereby their intestinal contents and fecal output become more concentrated (Figures 4A and 4C and Figure S4D), and they frequently excrete a subpopulation of oblong, even more concentrated deposits (referred to as reproductive oblong deposits or RODs, Figures 4A and 4D). These intestinal changes result from the action of the male-derived sex peptide (Liu and Kubli, 2003) on its neural receptor in the female, as revealed by the reduced ROD production resulting from neuronal downregulation of the sex peptide receptor (Figure S4G). Additional experiments in which sterile *ovo^D1^* or *egalitarian* mutant females were mated to wild-type or sex peptide mutant males further confirmed a requirement for the sex peptide in these changes in fluid balance, and established that they are uncoupled from egg production (Figures 4E, 4G, and 4H, and Figures S5B and S5C) or food intake (Carvalho et al., 2006; Liu and Kubli, 2003), given that *ovo^D1^* females do not increase their food intake after mating (Barnes et al., 2008). Instead, they appear to fulfill a water-preserving function, because they can be independently modulated by dietary water: virgin females (and males, albeit less frequently) can excrete RODs under conditions of reduced fluid availability (Figures S4E and S4F, and Figure 2I).

**Figure 5:**
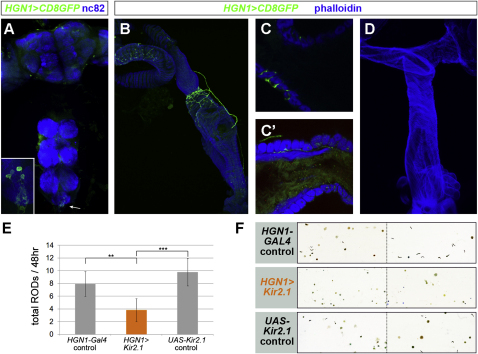
Enteric Neurons Required for the Sex Peptide-Induced Changes in Intestinal Physiology (A) *HGN1-Gal4* expression in the central nervous system. Note the cell bodies in the posterior tip of the ventral ganglion (arrow and inset), with axons exiting in the posterior nerve toward the hindgut. nc82 highlights the brain neuropil. (B) Innervation of the anterior hindgut, rectal valve, ampulla, and rectum by HGN1 axons. Muscles are labeled in blue with phalloidin. (C and C′) HGN1 neurites project through contiguous circular muscles toward the hindgut epithelium in the rectal valve (C), and toward the underlying layer of longitudinal muscle in the rectum (C′). (D) HGN1 axons do not innervate the female reproductive system. (E) Reduced ROD production in mated females following HGN1 neuron inactivation (p = 0.005 against *Gal4* control and p < 0.001 against *UAS* control, Mann-Whitney U test, n = 11–19 flies/set). (F) Representative fecal profiles of mated females with genetically inactivated HGN1 neurons and relevant controls. See the Supplemental Experimental Procedures for full genotypes. Graphs show average ± standard error of the mean.

To identify the neural effectors of these sex-peptide-triggered changes in fluid homeostasis, we selectively inactivated subsets of neurons by expressing the hyperpolarizing channel Kir2.1 from a collection of *Gal4* drivers. Reduced ROD production was observed upon silencing of *HGN1-Gal4* (Figures 5E and 5F), which only showed reproducible expression in a group of two to five hindgut-innervating neurons (Figures 5A and 5B). In addition to innervating the circular muscles or the rectal valve and rectum, these neurons innervate the epithelium underlying the rectal valve and the inner muscle layer of the rectum (Figures 5C and 5C′). Importantly, this line does not show expression in neurons innervating the female reproductive tract (Figure 5D).

Together, the results presented in this and the previous section indicate that, in addition to external nutrient availability, internal reproductive state impacts on postfeeding homeostatic processes involving pH and fluid balance in the intestine. Furthermore, they identify a group of epithelium-innervating intestinal neurons previously unknown in invertebrates as effectors of the intestinal changes in fluid homeostasis triggered by a reproductive hormone.

### The Digestive/Excretory System Responds to an Essential Vasopressin-like Systemic Signal to Maintain Fluid Homeostasis

**Figure 6:**
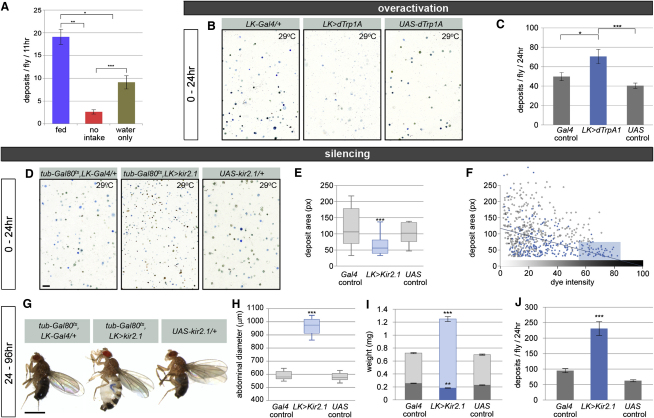
Central Diuretic Neurons Regulate Fluid Homeostasis (A) Water availability modulates a starvation-induced reduction in defecation rate. Both nutrient and nutrient/water deprivation lead to a reduction in defecation rate when compared to fed flies (p = 0.015 and p = 0.001, respectively, Student's t test, n = 8 flies/set), but the total number of excreta over a period of 11 hr is much smaller in the absence of nutrients and water than when water is available (p < 0.001, Student's t test, n = 8 flies/set). (B) Compared to controls, flies in which LK neurons have been overactivated for 24 hr produce lighter deposits (many of which are hardly visible). (C) Their defecation rate is higher than that of controls (p = 0.021 against *Gal4* control and p < 0.001 against *UAS* control, Student's t test, n = 17–20 flies/set). (D) Adult-specific inactivation of LK neurons results in smaller, more concentrated deposits than those excreted by wild-type controls (left and right panels). This phenotype is apparent after only 24 hr. The scale bar corresponds to 1 mm and applies to all three images. (E) Quantification of deposit size. Flies with inactive LK neurons excrete smaller deposits (p < 0.0001, Mann-Whitney U test). (F) LK neuron inactivation leads to the production of small, dark deposits. Size is inversely correlated to dye intensity in individual deposits excreted by *LK > Kir2.1* flies (blue trend line, p < 0.0001, linear model fit, displaying least-squares line) but not in either of the control groups (p = 0.19 for *Gal4* control, 0.17 for *UAS* control). Note the subpopulation of small and very dark deposits (shaded box) which is produced almost exclusively by *LK > Kir2.1* flies. (G) A longer period of LK neuron inactivation (4 days) leads to bloated flies. The scale bar corresponds to 1 mm and applies to all three images. (H) The abdominal diameter of these flies is much larger than that of control flies (p < 0.0001, Mann-Whitney U test, n = 24–30 flies/set). (I) Their average weight is much larger than that of the two controls (p < 0.001, Student's t test, n ≥ 24 flies/set). The weight difference is caused by fluid retention because the dry weights of all three genotypes, represented by the dark portions of all three bars, are comparable. In fact, the dry weight of LK-silenced flies is marginally lower (p = 0.001 or lower, Student's t test). (J) The defecation rate of flies in which LK neurons have been inactivated for 4 days is higher than that of control flies (p < 0.0001, Mann-Whitney U test, n = 10 flies/set). See the Supplemental Experimental Procedures for full genotypes. See also Figure S6 and Movie S1. Graphs show average ± standard error of the mean.

The dietary modulation of aspects of fluid homeostasis other than ROD production (namely, fluid content and intestinal emptying rate, Figures 2I and 6A, respectively) suggested additional mechanisms of fluid control. We hypothesized that the insect hormone leucokinin (LK), which can stimulate fluid secretion in vitro (O'Donnell et al., 1998) and is known to be released into the circulatory system by a small group of central neurons (Cantera and Nassel, 1992), may be relevant to the regulation of water homeostasis in vivo. We confined our genetic manipulations to adult male flies to bypass developmental effects (data not shown). Overactivation of LK neurons by expression of the heat-activated dTrpA1 ion channel resulted in increased diuresis, as revealed by the presence of lighter (less concentrated) and more abundant deposits (Figures 6B and 6C). By contrast, the genetic silencing of LK neurons elicited by expression of Kir2.1 (confined to adults using a *tubulin-Gal80ts* transgene) resulted in concentrated deposits of much smaller size (Figures 6D–6F), which were already apparent 24 hr after inactivation. Persistent inactivation over a longer period of time doubled defecation rate (Figure 6J), which we interpret as a compensatory mechanism to attempt to excrete excess fluid. Consistent with this idea, flies became extremely bloated and had highly enlarged abdomens (Figures 6G and 6H). Unlike the bloating resulting from a very full crop reported for leucokinin hypomorphic mutants after starvation (Al-Anzi et al., 2010), the swelling that we observed in fully fed, humid conditions is caused by excessive fluid retention outside the gut, as revealed by dry/wet weight experiments and examination of abdominal contents (Figure 6I and Movie S1), and can occasionally lead to abdominal rupture (data not shown).

LK has recently been shown to regulate meal size through its action on neuronal leucokinin receptors (Al-Anzi et al., 2010), but the additional expression of LK receptors in the renal tubules and the digestive tract (Radford et al., 2002; Veenstra et al., 2008) suggested a systemic mode of action in the regulation of fluid homeostasis. To test this idea, we selectively downregulated the expression of LK and its receptor in specific tissues using RNA interference (Figure S6). This confirmed that LK acts as a central neurohormone on its receptor in nonneural tissues to control fluid balance, in a manner analogous to mammalian vasopressin (Nussey and Whitehead, 2001).

Together, these findings indicate that visceral homeostasis in invertebrates is not only regulated by direct autonomic innervation but is also under the control of systemic signals emanating from the central nervous system.

### An Insulinergic Brain-Gut Neuronal Circuit Adjusts Feeding to Nutritional Conditions

**Figure 7:**
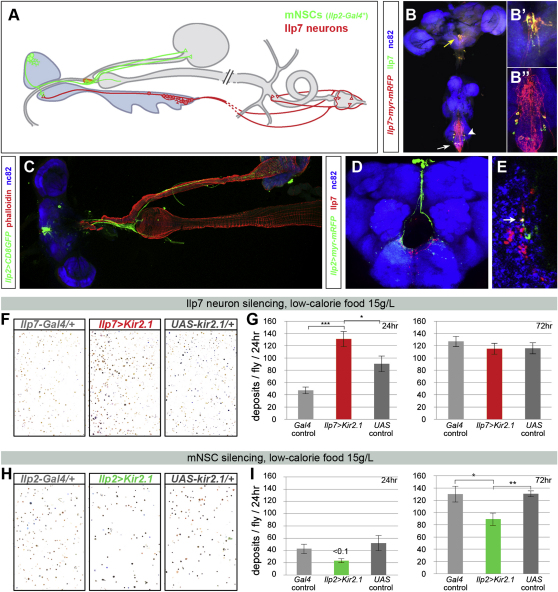
Differential Regulation of the Homeostatic Response to Nutrient Scarcity by a Brain-Gut Circuit of Insulin-Producing Neurons (A) Shown is connectivity of the two small subsets of central neurons expressing insulin-like peptides: the mNSCs, with cell bodies located in the protocerebrum, and Ilp7 neurons in the ventral ganglion. Cell bodies are depicted as circles. Dendritic endings are shown as branches, whereas axonal terminals are shown as empty triangles. (B–B″) Shown are Ilp7 neurons colabeled with an anti-Ilp7 antibody and a membrane-tagged *UAS-myr-mRFP* reporter. Two axonal fibers emanating from Ilp7 cell bodies in the ventral ganglion terminate in the subesophageal ganglion, as indicated by the abundance of vesicles positive for Ilp7 (B′). Note the profuse dendritic arborizations in the ventral ganglion (B″), which are relatively devoid of Ilp7 peptide (compare the strong green signal of B′ to B″). nc82 is used as a general neuropil marker in (B)–(E). (C) mNSC axons, labeled by their expression of a membrane-tagged reporter from *Ilp2-Gal4*, reach the ring gland, which in the adult is found at the junction between the stomach, esophagus, and crop duct. Additional nerves project along the crop duct and arborize on the crop muscles. (D) Dendrites emanating from the mNSCs (visualized by their expression of *UAS-myr-mRFP* from *Ilp2-Gal4*, displayed in green) reach the subesophageal ganglion where Ilp7 axons (labeled with anti-Ilp7 antibody and displayed in red) terminate. For the sake of consistency with other panels, the green and red channels have been inverted in (D) and (E). (E) Single, high-magnification optical slice showing one punctum coexpressing Ilp7, *Ilp2 > myr-mRFP* and the synaptic marker nc82, suggesting synaptic contact between Ilp7 axons and mNSC dendrites. (F) Increased fecal output of Ilp7 neuron-silenced flies relative to controls on a low-calorie diet for 48 hr. The shift to more orange hues occurs normally. (G) Time course analysis of defecation rate under dietary restriction. After 24 hr, only flies with genetically inactivated Ilp7 neurons have increased defecation rate (p < 0.0001 and p = 0.029, Mann-Whitney U test and Fisher's combined probability test of two experiments, n ≥ 28 flies/set. Graphs and statistics are displayed for one of the two experiments). After a longer period of nutrient deprivation (72 hr), there are no significant differences between the defecation rate of controls and Ilp7 neuron-silenced flies. We could not measure food intake directly using the CAFE assay due to the high lethality associated with this food dilution when supplied in liquid form, but we confirmed that the increased fecal output results from increased food intake, because it is not accompanied by reduced gut capacity (Figure S7A) or a decrease in the dye content of excreta (Figure 7F and Figure S7C). (H) Fecal outputs of control and mNSC-silenced flies fed a low-calorie diet for 48 hr. Inactivation of mNSC neurons was confined to the late third-instar stage onward using a late *Gal4* driver to circumvent defects associated with reduced growth during development. Flies with genetically inactivated mNSCs excrete fewer deposits. The shift to more orange hues occurs normally. (I) Time course analysis of defecation rate following the nutritional challenge. After 24 hr, the defecation rate of mNSC-silenced flies tends to be lower than that of control flies (p < 0.001 and p = 0.23, Mann-Whitney U test and Fisher's combined probability test of two experiments, n ≥ 28 flies/set). After 72 hr, their defecation rate becomes significantly lower than that of controls (p < 0.0001 and p = 0.002, Mann-Whitney U test and Fisher's combined probability test of two experiments, n ≥ 28 flies/set). Graphs and statistics are displayed for one of the two experiments. As in the case of mNSC inactivation, intestinal capacity and dye content controls confirmed that these changes are reflective of food intake (Figures S7B, S7D, and 7H). See the Supplemental Experimental Procedures for full genotypes. See also Figure S7. Graphs show average ± standard error of the mean.

Having established that intestinal homeostasis is subject to neuronal and systemic regulation, we wondered whether the role of intestinal neurons can extend beyond their local action to affect behaviors related to nutrient intake or processing. Our previous work had established that about half of the Ilp7 neurons, a small group of 16–20 insulinergic neurons with cell bodies located in the ventral ganglion (analogous to the spinal cord), innervate the adult hindgut (pylorus and rectal ampulla, Figure 7A and Brogiolo et al., 2001; Miguel-Aliaga et al., 2008). Inactivation of Ilp7 neurons had no apparent effect under normal feeding conditions (data not shown). However, we observed that flies with Ilp7-silenced neurons overreact to poor nutritional conditions by increasing their food intake faster than control flies (Figures 7F and 7G, additional controls in Figures S7A and S7C; males were used to circumvent phenotypes secondary to egg production).

A subpopulation of Ilp7 neurons does not innervate the hindgut (Miguel-Aliaga et al., 2008), thus raising the possibility that the observed effect on food intake is not related to intestinal innervation. Detailed examination of the neuroanatomy of Ilp7 neurons revealed that in the ventral ganglion, the dendrites of the hindgut-innervating Ilp7 neurons (Figures 7B and 7B″, white arrow) are densely arborized with those of another subset of Ilp7 neurons located in more anterior segments (Figures 7B and 7B″, arrowhead). The axonal terminals of these neurons (Figures 7B and B′, yellow arrow) appear to make direct synaptic contact with dendrites emanating from the small group of 10–14 brain median neurosecretory cells (mNSCs, Figures 7D and 7E) which express three different insulin-like peptides (Broughton et al., 2005) and, in the adult, also innervate the intestine further anteriorly (Figures 7A and 7C; Cao and Brown, 2001). In contrast to Ilp7 neuron inactivation, mNSC silencing led to a persistent hypophagic response to nutrient scarcity, as indicated by their reduced fecal output (Figures 7H and 7I, additional controls in Figures S7B and S7D).

Together, our results identify a neuronal circuit consisting of two groups of insulin-producing neurons, which have release sites on all three innervated intestinal domains and function to adjust feeding in response to nutrient scarcity. Anatomical analysis of their neuronal connectivity suggests that the differential modulation of food intake by these two populations may be achieved, at least partly, by synaptic modulation of mNSCs by Ilp7 neurons.

## Discussion

Our work has uncovered a central role for the intestine in the execution of extensive (and previously uncharacterized in invertebrates) homeostatic regulation subsequent to the decision whether or not to eat, and has identified intestinal neurons and systemic signals as key mediators of the crosstalk between internal organs. The implications of our findings are discussed below.

### Between What Goes in and What Comes Out: Nutrition, Reproduction, Longevity, and the Intestine

Our experiments provide evidence for significant regulation of nutrient utilization independent of intake. For example, we find intestinal transit to be differentially modulated by a low-calorie diet and reproductive state: two conditions known to increase food intake. In contrast to the faster emptying rate associated with a low-calorie diet (Figure 1M), the action of a reproductive hormone (the sex peptide) leads to concentration of intestinal contents and slower intestinal transit in mated females (Figures 4A–4D and Figure S4D). This effect is strikingly similar to that of progesterone, oxytocin, and estrogen on intestinal passage, secretion, and water absorption, which cause bloating and constipation during pregnancy (Keller et al., 2008). These reproductive gastrointestinal changes may be associated with enhanced nutrient absorption: a possible competitive advantage at a time of high nutritional demands.

The differential enteric physiology of mated females and diet-restricted flies also points to a link between internal diuresis and life span, whereby longevity would be associated with faster intestinal transit and/or diets with a higher water/calorie ratio. This would explain the recently reported deleterious effects of water-poor dietary regimes (Ja et al., 2009), and why the impact on life span of dietary restriction (positive) and mating (deleterious in females) is not entirely attributable to calorie intake or egg production (Barnes et al., 2008; Mair et al., 2005). Consistent with this idea, we also find that sterile *ovo^D1^* mutant females, which do not increase their long-term food intake after mating but still experience mating survival costs (Barnes et al., 2008), also concentrate excreta (Figures 4E and 4G). It will be interesting to establish how intestinal physiology is affected by the amino acid imbalance recently found to account for the life-shortening effects of certain diets (Grandison et al., 2009).

Finally, it will be instructive to investigate how intestinal flora affects or is affected by the reproductive changes in enteric physiology triggered by mating, diet, and internal metabolic state. Increasing evidence points to a differential role for specific phyla of gut bacteria in nutrient acquisition, energy regulation, and obesity (Turnbaugh et al., 2009). Given the relative simplicity of the intestinal microbial consortium of lab-reared *Drosophila* strains (Cox and Gilmore, 2007), our behavioral and physiological readouts could easily be exploited to investigate the interactions between this bacterial diversity and organismal homeostasis.

### Intestinal Neurons, Feeding, and Metabolism

The organizational principles of mammalian enteric nervous systems are broadly conserved in *Drosophila*. In contrast to the gut of nematode worms (which is devoid of direct innervation, Avery and Thomas, 1997), we find the *Drosophila* intestine to be extensively innervated by sensory and efferent fibers confined to three discrete portions containing smooth muscle sphincters or valves: a neural architecture suggestive of “checkpoints” where intestinal transit may be sensed and modulated. Interestingly, we have also observed that a subset of enteric neurons innervate the underlying intestinal epithelium, and have found that at least one such lineage effects changes in water balance associated with reproduction. This kind of innervation has not been previously described in invertebrates, and is reminiscent of the fibers of the submucosal plexus, which regulate epithelial crypt cell secretion in mammals (Furness, 2006).

The direct innervation of the adult intestine by insulinergic fibers suggests a novel mode of action for Ilps. Although Ilp2 appears to act systemically to regulate larval growth (Arquier et al., 2008; Geminard et al., 2009), endogenous Ilps have not so far been detected in the circulation. Hence, it is possible that Ilps modulate intestinal physiology locally to regulate food intake (and perhaps some of the previously reported mNSC functions in glucose homeostasis and energy storage, Broughton et al., 2005; Rulifson et al., 2002). In humans and other mammals, neural and hormonal signals originating from the intestine can promote satiety and modulate pancreatic insulin secretion (Das, 2010; Strader and Woods, 2005). Insulin could, in turn, convey information about nutritional state to the intestine, which would integrate additional signals (such as those that we have found to emanate from reproductive tissues or diuretic centers) to regulate nutrient processing or the production of intestinal satiety signals. Such intestinal roles would be consistent with the finding of isolated insulin-producing secretory cells in the digestive tract of other invertebrates and protochordates (Sherwood et al., 2005). In this regard, the positioning of the ring gland (which is profusely innervated by mNSC fibers) in close proximity to the esophagus and anterior midgut of adult flies (Figures 7A and 7C) is strikingly reminiscent of the islet organ of primitive vertebrates such as hagfish: a discrete aggregation of insulin-producing cells found in the same anatomical location (Jorgensen et al., 1998). Hence, the acquisition of insulinergic fate by endocrine organs of different evolutionary origin (Jorgensen et al., 1998; Wang et al., 2007) may reflect a shared requirement for a local source of insulin release close to the intestine. In any event, it suggests that a differential developmental origin (brain and gut insulins) does not necessarily imply functional diversification.

### Neural Control of Internal Fluid Homeostasis

We have uncovered an essential role for a very restricted group of central neuroendocrine cells in the systemic regulation of diuresis: a role strikingly similar to the effect on the kidney of vasopressin, a hormone synthesized in the hypothalamus and released from the pituitary gland into the blood stream (Nussey and Whitehead, 2001). Loss of LK signaling has acute effects: fluid retention is such that adult wet weight almost doubles within a few days (Figure 6I), eventually leading to death. This effect contrasts with the relatively modest weight alterations resulting from interfering with adult feeding, energy storage, or developmental growth (for example, see Lee et al., 2004; Okamoto et al., 2009). The importance of fluid regulation, both during development and in adult homeostasis, may therefore have been underestimated. The connections between water consumption, energy intake, and body weight in humans are poorly understood (Yao and Roberts, 2001). Our work and a recent study (Al-Anzi et al., 2010) point to a model wherein one neurohormone (LK) acts on the same receptor centrally to regulate food intake and peripherally to maintain fluid balance.

The recent discovery of axonal terminals emanating from water-sensing neurons in the subesophageal ganglion (Cameron et al., 2010), where leucokinin-positive dendrites arborize (de Haro et al., 2010), suggests sensory input into this vasopressin-like system. Phenotypes like the one resulting from LK neuron silencing provide a behavioral readout with which to test this idea or investigate the nature of possible internal osmolality sensors.

### A Metabolic Behavior in an Invertebrate

In *C. elegans*, the genetic analysis of defecation rate has proved to be an excellent system with which to identify developmental or metabolic genes (Branicky and Hekimi, 2006). Our assay allows quantification of additional aspects of diuresis, enteric function, and food intake. It is thus the first integrative behavioral readout for metabolism in an invertebrate, which can be used in high-throughput screens for genes or compounds regulating diuresis, gastrointestinal physiology, ion transport, and their neural, nutritional, and reproductive control. In particular, having uncovered at least two distinct mechanisms of nutritional modulation of acid-base homeostasis (only one of which is *foxo* dependent), it will now be of interest to use the pH of excreta as a readout for genetic screens aimed at identifying the metabolic pathways involved, the intestinal mechanisms of pH regulation, and the contribution and site of action of the large number of previously reported *foxo* targets (Teleman et al., 2008). In parallel, our assay will also enable future studies aimed at establishing the contribution of specific neurons, peptides, and cell populations, such as gut stem cells and enteroendocrine cells, to gastrointestinal function and organismal homeostasis.

## Experimental Procedures

### Defecation and Intestinal Assays using Food Dyes

After boiling, food was allowed to cool down to 60°C–65°C before it was supplemented with 0.5% Bromophenol blue sodium salt (B5525, Sigma), 0.5% Bromocresol purple sodium salt (17492, Sigma), and/or 0.03% Fluoropoo (4,4-difluoro-1,3,5,7,8-pentamethyl-4-bora-3a,4a-diaza-s-indacene, BODIPY 493/503, Invitrogen). All three dyes were found to be nontoxic to flies and to remain in the digestive tract until they were excreted. Neither BPB nor Fluoropoo was found to affect food intake (measured as ingested volume using the CAFE assay after a 24 hr acclimation period, data not shown). Dissections of dye-containing intestines were performed in saline, leaving the head and posterior cuticle intact to prevent dye leak. Defecation rate experiments and ROD quantification experiments were performed on 10–30 flies, which were individually placed in clear plastic cuvettes (UV grade PPMA cuvettes, Kartell) containing 0.5 ml of food supplemented with BPB (alone or in combination with Fluoropoo). Analyses of the nature of excreta (including color, shape, size, and dye intensity) were conducted as above for individual flies, or in 50 mm petri dishes (Sterilin) containing food supplemented with BPB and groups of eight to ten flies. Digital images of petri lids or cuvette walls were obtained using an Epson Perfection 4990 Photo scanner. Whole-image background adjustment was applied using Adobe Photoshop.

### Quantitative Analysis of Defecation Behavior

Quantification of excreta was performed manually using cuvettes housing single flies. Digital scans were analyzed for qualitative traits such as color or shape features using Volocity 64 software (Improvision). Briefly, deposits were automatically identified through a saturation and intensity filter (Find Objects Using HSI), corrected for noise and presence of holes (Remove Noise From Objects, Fill Holes in Objects), and filtered by size. The specific filtering values applied were adapted to each experimental scan, but were always identical for all compared samples. Manual correction was employed to remove false positives (e.g., food debris), add individual deposits not identified by the filter, and correct software misinterpretations (e.g., overlapping deposits). Color (Mean Red, Mean Green and Mean Blue) and size (Pixel Count and Perimeter) values were exported to the R environment (R Development Core Team, 2009) and converted to Hue, Saturation, Lightness values according to previously described formulas (Agoston, 2005). Dye intensity was calculated as 100 − lightness. Hue density graphs display the population distribution of the hue variable as a continuous estimate smoothed by a Gaussian kernel (R density function, bandwidth = 7, n ≥ 130 deposits per set in all graphs shown, except for Figure 3D, in which bandwidth = 10, n ≥ 50).

### Metabolic and Mating Assays

For starvation experiments, single flies reared in standard food supplemented with BPB and Fluoropoo were transferred to cuvettes containing either the same food, water-soaked filter paper, or nothing. In dietary restriction experiments, single flies reared in standard food supplemented with BPB were transferred to cuvettes containing either a nutritious diet (5.4% sucrose, 3.6% autolysed yeast flakes, 0.5% BPB, 0.18% Nipagin) or food with low nutritional value (0.9% sucrose, 0.6% autolysed yeast flakes, 0.5% BPB, 0.18% Nipagin). After the desired time had elapsed, cuvettes were emptied by transferring the single flies to new cuvettes and deposits were counted.

#### Quantification of Gut Contents

Quantification of gut contents was conducted by homogenizing sets of three flies in 30 μl of water using a plastic pestle in a 1.5 ml Eppendorf tube, followed by 2′ centrifugation at 14,680 rpm, which was repeated for a further 2′ after collecting the first supernatant. The dye content of the second supernatant was determined as the absorbance at 594 nm using a NanoDrop ND-1000 Spectrophotometer. Background absorbance was obtained by processing sets of flies reared on dye-free food in the same manner.

#### Wet/Dry Weight Experiments

Wet/dry weight experiments were carried out by drying sets of five flies overnight at 50°C over a layer of silica gel and weighing them before and after the procedure on a Mettler-Toledo XS105 precision balance.

#### Measurements of Abdomen Size

Flies used for measurements of abdomen size were photographed with a Leica DFC 420C camera attached to a Leica MZ16F dissection microscope at 10× magnification. Leica Acquisition Software was used to quantify the width of the abdomen between the second and third abdominal segment.

#### pH Assays

When used for pH experiments, standard food was adjusted to specific pHs with defined volumes of HCl or NaOH. Final pH was assessed using pH indicator paper (Whatman, type CF). The pH of the defined food used to test the effect of dietary restriction and food composition was monitored using a Mettler Delta 340 pH meter and was brought to a pH of 5.4–5.5 using HCl or NaOH if necessary, so as to ensure that the final pH of all the different diets was the same regardless of their food composition. Two different pH indicators were used because of their convenient color range (pH 3.0–4.6 for BPB, pH 5.2–6.8 for Bromocresol purple).

#### Mating Experiments

Female flies marked as mated were reared in the presence of the opposite sex until the time of the experiment. Mating was subsequently confirmed by the presence of larvae in the food. Males and females were allowed to mate once over a 3 hr period (in the case of the *ovo^D1^* and *egl* experiments) or overnight for the *SPR-RNAi* experiments.

Extended experimental procedures and fly stock information are available in the Supplemental Information.

## Supplemental Information

Document S1. Seven Figures, One Table, Supplemental Experimental Procedures, and Supplemental References

Movie S1. Increased Fluid Retention upon Genetic Inactivation of LK Neurons Related to Figure 6. Insertion of the end of a pair of forceps in the abdomen of a bloated LK-silenced fly in PBS reveals that swelling is caused by fluid retention; note the resulting deflation and the absence of air bubbles.
