# Enteric Neurons and Systemic Signals Couple Nutritional and Reproductive Status with Intestinal Homeostasis

**DOI:** 10.1016/j.cmet.2011.12.010

**Published:** 2012-01-04

**Authors:** Paola Cognigni, Andrew P. Bailey, Irene Miguel-Aliaga

**Affiliations:** 1 Department of Zoology, University of Cambridge, Downing Street, Cambridge CB2 3EJ, UK; 2 Division of Developmental Neurobiology, MRC National Institute for Medical Research, The Ridgeway, Mill Hill, London NW7 1AA, UK

(Cell Metabolism *13*, 92–104; January 5, 2011)

In the Experimental Procedures of the above paper (under “Defecation and Intestinal Assays using Food Dyes”), the long chemical name for Fluoropoo (CholEsteryl BODIPY FL C_12_) was incorrectly stated as that of BODIPY alone. The correct name and source are as follows: CholEsteryl 4,4-Difluoro-5,7-Dimethyl-4-Bora-3a,4a-Diaza-*s*-Indacene-3-Dodecanoate (CholEsteryl BODIPY FL C_12_), Invitrogen (catalog number C-3927MP).

Although this mistake does not change any of the results or conclusions of the study, the authors regret any confusion it may have caused.

