# Supplementary material for: Enteric Neurons and Systemic Signals Couple Nutritional and Reproductive Status with Intestinal Homeostasis
Source: Cell Metab. 2011 Jan 5;13(1):92–104. doi: 10.1016/j.cmet.2010.12.010 (PMC3038267; doi:10.1016/j.cmet.2010.12.010)
Supplement: Document S1. Seven Figures, One Table, Supplemental Experimental Procedures, and Supplemental References [file mmc1.pdf]

Cell Metabolism, Volume *13*

Supplemental Information

Enteric Neurons and Systemic Signals Couple Nutritional and Reproductive Status with Intestinal Homeostasis

Paola Cognigni, Andrew P. Bailey, and Irene Miguel-Aliaga

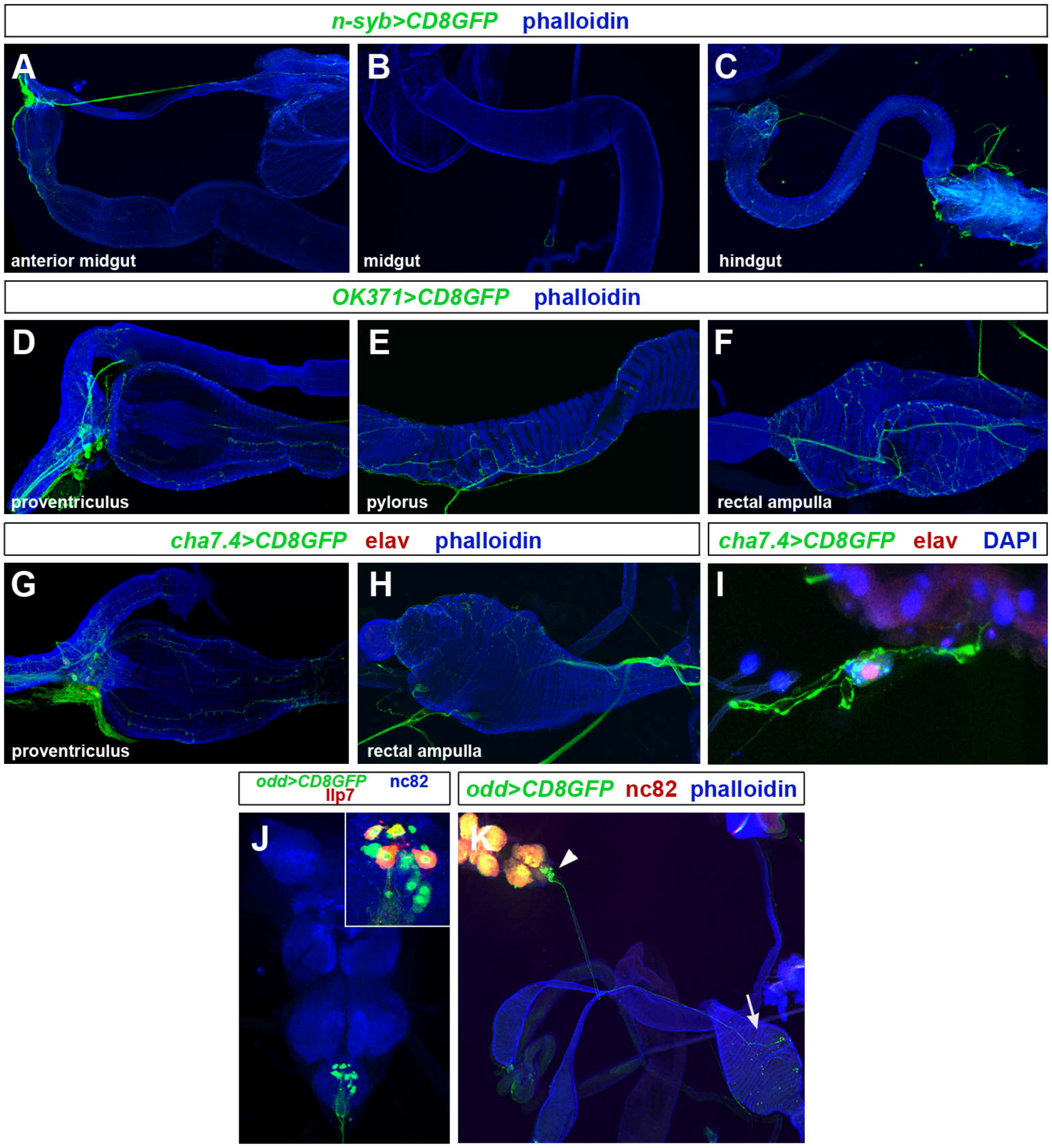

Figure S1. Innervation of the *Drosophila* intestine. Related to Figure 1.
(A-C) A membrane-tagged GFP expressed from the pan-neuronal driver *n-syb-Gal4* confirms the sparse innervation of the adult intestine. (A) Innervation of the oesophagus, crop and anterior midgut (proventriculus). (B) No *n-syb-Gal4*-positive neurites are apparent in the middle portion of the midgut. (C) In the hindgut, *n-syb-Gal4* neurites innervate the pylorus and rectal ampulla. This sparse innervation was additionally confirmed by staining with a mouse anti-elav antibody (Figures 1I, S1G-I and data not shown). (We note that the rat anti-elav antibody labels visceral muscle nuclei in addition to neurons).
(D-F) Glutamatergic innervation of the proventriculus (D), pylorus (E) and rectal ampulla (F), as revealed by expression of a membrane-tagged GFP from the *OK371-Gal4* driver.
(G-H) A subset of the peripheral neurons on the proventriculus (G) and hindgut sensory neurons (H, both labelled with anti-elav antibody) are cholinergic, as revealed by their expression of *cha7.4-Gal4*.
In A to H, smooth muscles are visualized with phalloidin (in blue). Anterior (oral) is to the left in all images.
(I) High magnification image of one of the hindgut-innervating cholinergic sensory neurons shown in H. The nucleus (labelled with DAPI) is elav-positive.
(J) The cell bodies of neurons positive for *odd-Gal4*, which label the insulin-producing Ilp7 neurons and at least one other lineage (inset), are located in the posterior segments of the ventral ganglion. nc82 is used as a general neuropil marker.
(K) *odd-Gal4*-positive neurons (arrowhead) send out long axons (visualized in green with a membrane-tagged GFP) which innervate the hindgut (labelled in blue with phalloidin, arrow) directly. nc82 (in red) is used to highlight the ventral ganglion.

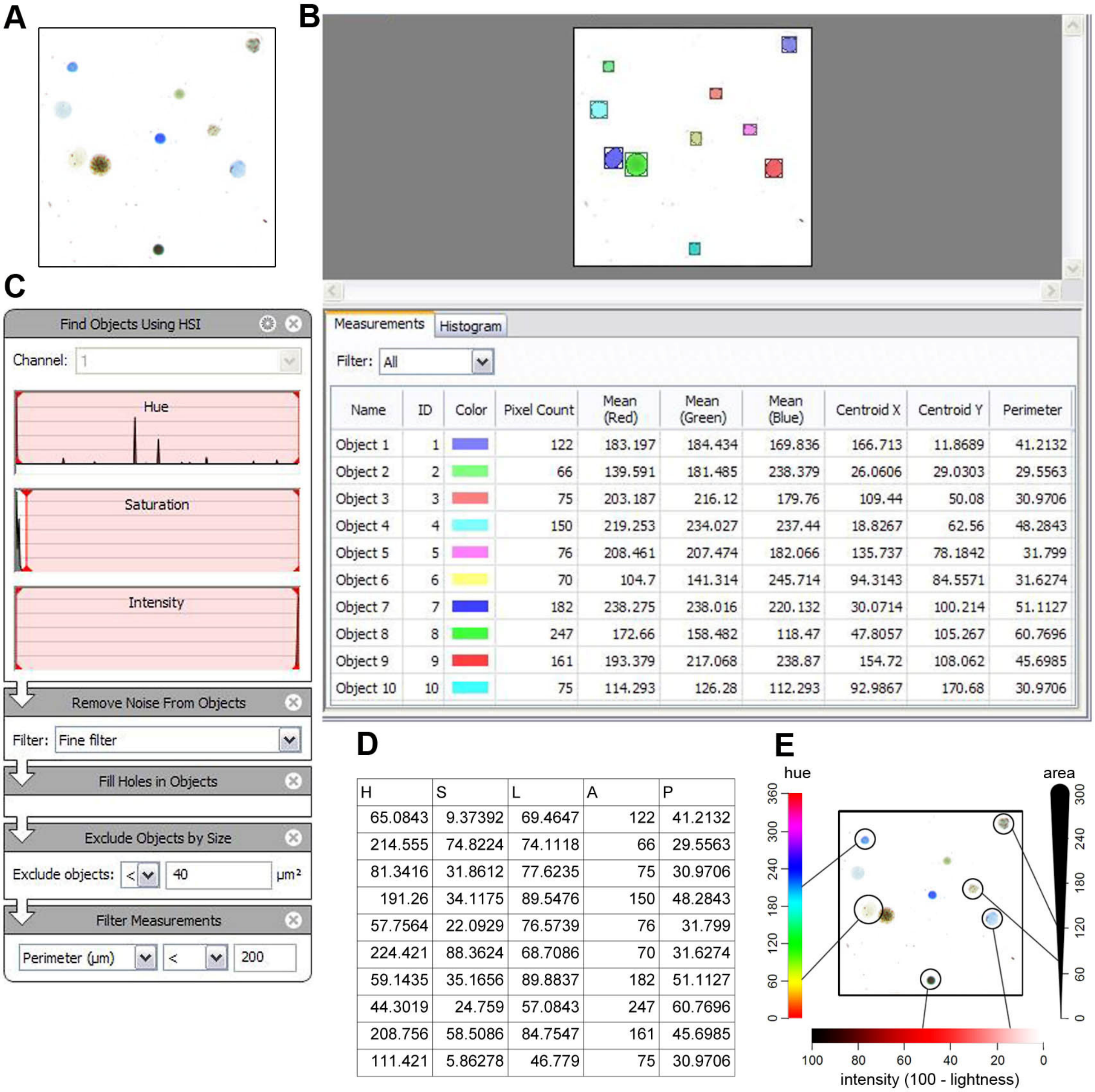

Figure S2. Quantitative analysis of faecal output. Related to Figure 2.
(A) Raw data: a scan of the deposits of BPB-fed flies on clear plastic.
(B) Volocity identifies single deposits and computes their average RGB value, position and perimeter (single deposits are arbitrarily coloured for clarity).
(C) Volocity protocol and filters used for deposit identification.
(D) Conversion of Volocity measurements to hue (H), saturation (S), lightness (L), area (A) and perimeter (P).
(E) Example of hue, lightness and size values for specific deposits relative to defined ranges. When applied to an entire population, this analysis generates density plots which reflect population distributions for a particular value (see, for example, Figure 2H).

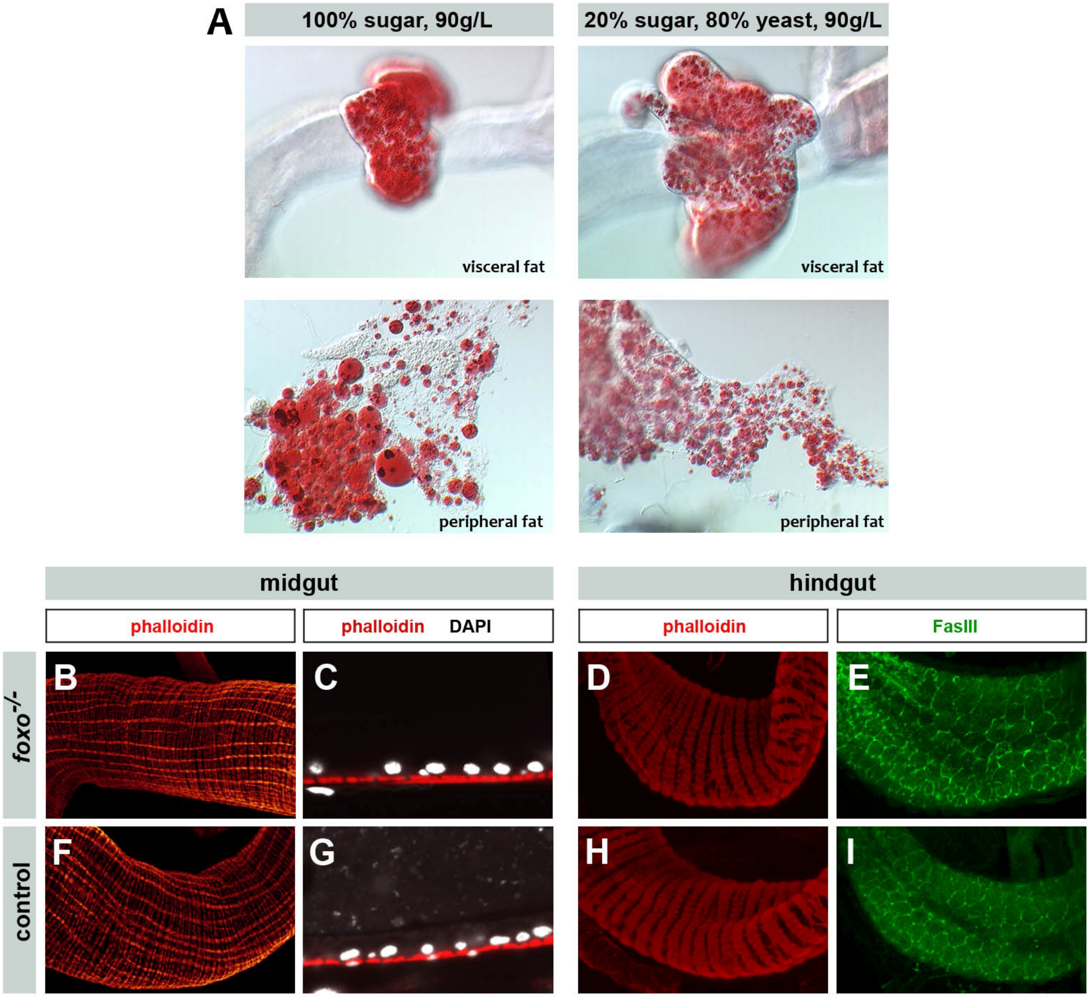

Figure S3. Metabolic changes triggered by diet and gut morphology of *foxo* mutants. Related to Figure 3.
(A) The size of lipid droplets in the fat body of sugar-fed flies is larger than in flies fed a yeast-rich diet. This is suggestive of diet-induced changes in internal metabolism.
(B) Detail of circular and longitudinal midgut muscles of $foxo^{21}/foxo^{Df(3R)Exel8159}$ mutants, stained with phalloidin. No obvious defects are observed.
(C) Longitudinal slice of a $foxo^{21}/foxo^{Df(3R)Exel8159}$ mutant midgut. DAPI staining reveals the presence of large endoreplicating nuclei (enteroyctes), and small nuclei corresponding to intestinal stem cells/enteroendocrine cells.
(D) Detail of the circular muscles of the hindgut of a $foxo^{21}/foxo^{25}$ mutant, stained in red with phalloidin. No abnormalities are apparent.
(E) No morphological defects are apparent in the hindgut epithelium of a $foxo^{21}/foxo^{25}$ mutant, as revealed by FasIII staining.
(F-I) w^{1118} control intestines stained as the panels above.

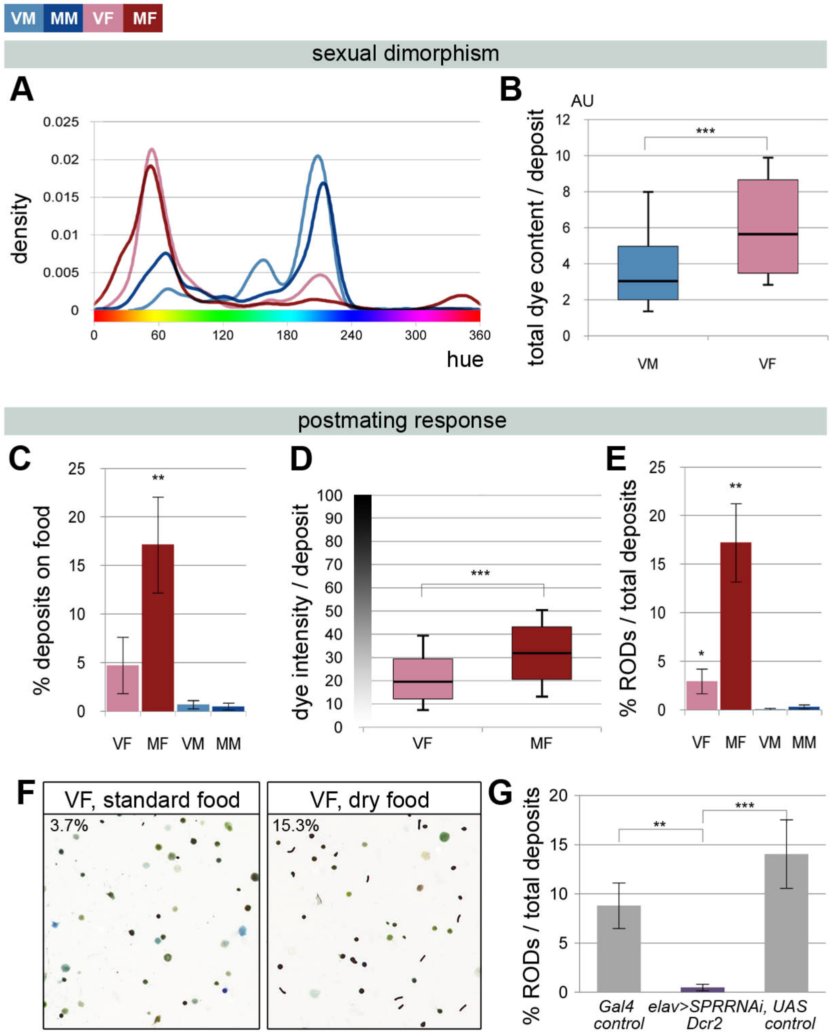

Figure S4. Effects of reproductive state on intestinal physiology. Related to Figure 4.
All graphs are colour-coded according to sex and mating status. Abbreviations are: VM, virgin male; MM, mated male; VF, virgin female; MF, mated female. All experiments in this Figure were conducted with groups of 9-12 (A to F) or 9-14 (G) flies.
(A) Metabolic differences between males and females, as revealed by the differential colour distribution of their deposits. Female excreta are predominantly more acidic, with a large proportion clustering around green/yellow hues. By contrast, males produce a larger percentage of blue, more basic deposits ($p < 0.0001$, two-sample Kolmogorov-Smirnov test).
(B) Males excrete less than females in each defecation event, as indicated by their reduced total dye content (dye intensity x area) per deposit ($p < 0.0001$, Mann-Whitney *U* test).
(C) Increased frequency of defecation events on food in females upon mating ($p < 0.003$, MF versus VF, Mann-Whitney U test). This is likely to result from their increased egg laying activity.
(D) The deposits of mated females are more concentrated (as shown by their dye intensity value) than those of virgin females ($p < 0.0001$, Mann-Whitney *U* test).
(E) RODs are preferentially excreted by mated females ($p = 0.003$ or lower versus VF or males, Mann-Whitney *U* test). They can be produced by virgin females, albeit at a reduced frequency ($p = 0.028$ or lower versus males, Mann-Whitney *U* test). Excretion of RODs by males is possible but extremely rare.
(F) Representative faecal output of virgin females under normal conditions (left panel) and after feeding on dry food (standard food dried at $50^{\circ}C$ for 2h, right panel). Note the increased frequency of ROD production (15.3% vs. 3.7%).
(G) Females in which the sex peptide receptor has been downregulated specifically in the nervous system do not produce as many RODs after mating to wild-types males as control females do ($p < 0.001$ against *UAS* control, $p = 0.005$ against *Gal4* control, Mann-Whitney *U* test). Full genotypes are: w^{1118}, *elav-Gal4*/w^{1118}; +; +; + (*Gal4* control), w^{1118}, *UAS-Dcr2*/ w^{1118}, *elav-Gal4; UAS-SPR-RNAi/+; +; +* (*elav>SPR-RNAi, Dcr2*) and w^{1118}, *UAS-Dcr2*/ w^{1118}; *UAS-SPR-RNAi; +; +* (*UAS* control).

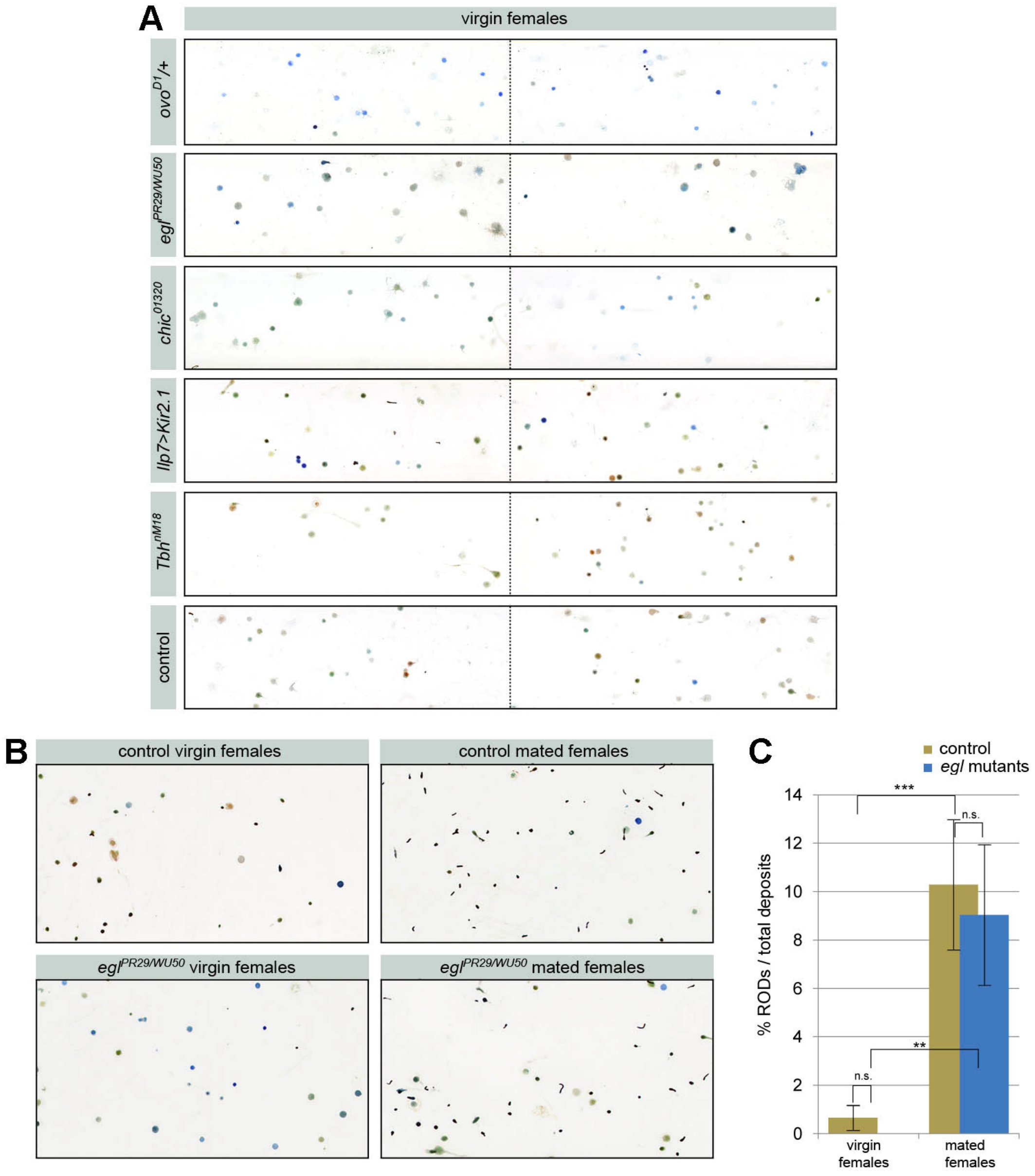

Figure S5. Intestinal acid-base and fluid balance in sterile mutant females. Related to Figure 4.
(A) The deposits of *ovo*^{D1}/+ virgin females (in which oogenesis is blocked before stage 4 and ovaries with no or few egg chambers degenerate before vitellogenesis (Busson et al., 1983; Perrimon and Gans, 1983)), are much more basic than those of control virgin females, as revealed by their bluer hue. The deposits of *egl* or *chic*^{01320} mutant virgin females (in which the egg chambers are formed but no oocyte is produced due to oocyte misspecification or incomplete transfer of nurse cell cytoplasm, respectively (Cooley et al., 1992; Schupbach and Wieschaus, 1991; Verheyen and Cooley, 1994), are more basic than those of control virgin females, but less than those of *ovo*^{D1}/+ virgin females. By contrast, the deposits of Ilp7-silenced or octopamine-less *Tbh* mutant virgin females, in which eggs are produced but are jammed in the oviduct (Monastirioti, 2003; Yang et al., 2008), are comparable to those of controls. Experiments were conducted using standard, nutritious food to prevent food intake phenotypes resulting from Ilp7 neuron inactivation (see Figure 7). Together, these phenotypes indicate that the sexual dimorphism in intestinal pH is caused by egg production and not by egg laying.
(B) Like wild-type flies, sterile *egl* mutant females increase their production of RODs after mating. This confirms that the production of concentrated deposits triggered by mating is not secondary to increased egg production or egg laying.
(C) Quantification of ROD production. Similar to wild-type females (yellow bars), mating increases the relative frequency of concentrated RODs in *egl* mutant females (blue bars, $p < 0.001$ for control flies and $p = 0.002$ for *egl* mutants, Mann-Whitney *U* test, $n = 10$ flies for each genotype/condition). The response of both genotypes to mating is quantitatively equivalent ($p > 0.1$).

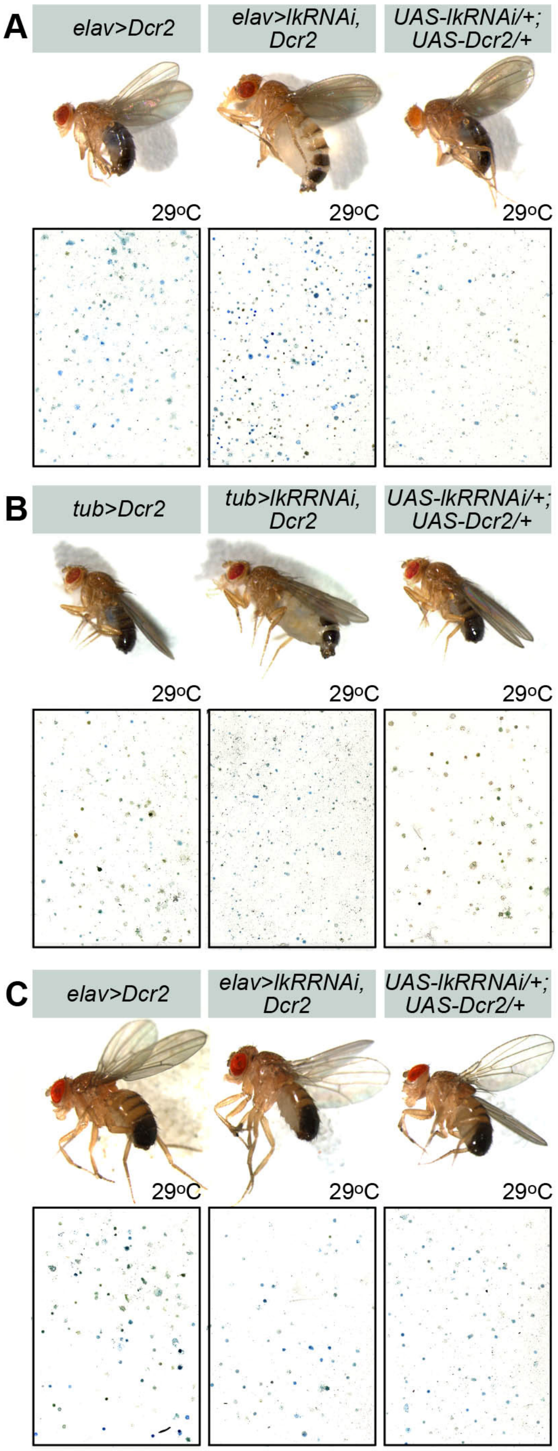

Figure S6. Regulation of fluid homeostasis by neuronal LK and peripheral LK receptor. Related to Figure 6.
(A) Downregulation of LK expression in the nervous system using the pan-neuronal driver *elav-Gal4* recapitulates the effects of LK neuron inactivation: flies become bloated, and they produce more deposits of smaller size than those of control flies.
(B) Ubiquitous downregulation of the LK receptor leads to identical phenotypes.
(C) When downregulation of the LK receptor is confined to neural tissues, flies are indistinguishable from control flies, indicating that the fluid retention phenotypes observed in (B) result from LK receptor downregulation in non-neuronal tissues (namely, the digestive and/or excretory systems).

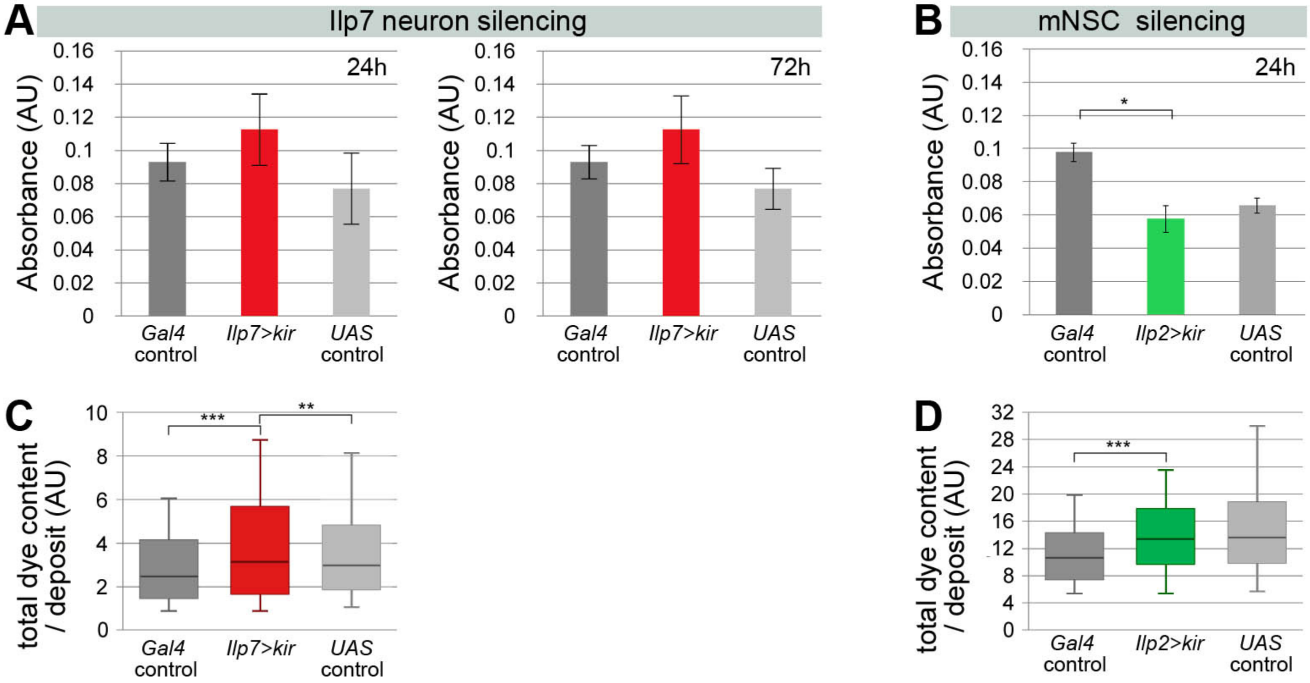

Figure S7. Controls for the food intake phenotypes of Ilp7- and mNSC-silenced flies on a low-calorie diet. Related to Figure 7.

(A) Intestinal contents (estimated by the absorbance of BPB, see Experimental Procedures) of Ilp7-silenced flies after 24 or 72h of feeding on a low-calorie diet (15g/L). The internal dye content of flies with genetically silenced Ilp7 neurons is not lower than that of control flies, indicating that their increased faecal output after 24 hours is not secondary to their inability to accumulate food in their intestine (not significant, Student's *t*-test and Fisher's combined probability test of two experiments, $n \geq 28$ flies for each genotype. Graphs and statistics are displayed for one of the two experiments).

(B) The internal capacity of flies with genetically silenced mNSCs neurons is not higher than that of control flies after 72h of feeding on the low-calorie diet, indicating that their reduced faecal output at this time point is not caused by food retention and/or a larger intestinal capacity ($p = 0.05$ against *Gal4* control and not significant against *UAS* control, same statistical analysis as above).

(C) The dye content of the deposits produced by flies with genetically silenced Ilp7 neurons on a low-calorie is not lower than that of controls (it is, in fact, significantly higher: $p < 0.0001$ against *Gal4* control, $p = 0.003$ against *UAS* control, two-sample Kolmogorov-Smirnov test, n = 8-10 flies for each genotype). A and C together indicate that the higher defecation rate of Ilp7-silenced flies is reflective of their higher food intake.

(D) The dye content of the deposits produced by mNSC-silenced flies on a low-calorie is in between that of controls. B and D together indicate that the lower defecation rate of Ilp2-silenced flies is reflective of their lower food intake ($p < 0.0001$ against *Gal4* control, not significant against *UAS* control, two-sample Kolmogorov-Smirnov test, n = 8-10 flies for each genotype).

Physiological parameter	Raw data	Display	Statistical analysis
pH	Hue	Density plot	Kolmogorov-Smirnov test
Fluid content	Intensity (100 - lightness)	Box plot	Mann-Whitney U test
Dye content	Intensity x Area	Box plot	Mann-Whitney U test
Internal gut capacity	BPB absorbance 592nm	Bar graph	Student's *t*-test
Food intake	Estimated as rate (only if controlled for dye content, internal capacity and steady-state dye accumulation)	Bar graph	Mann-Whitney U test

Table S1. Quantification of physiological parameters using defecation behaviour. Related to Figure 2.
The table indicates the raw data, as obtained by Volocity analysis of scanned images, required for the quantification of specific physiological parameters. It also states how data are displayed and the statistical analyses required for comparisons across genotypes or different conditions. Differences in food intake between genotypes can be inferred from differences in defecation rate only if:

1. The dye content per deposit of the genotypes is comparable, or it is different in the same direction as the difference in defecation rate (e.g. a genotype with higher defecation rate than controls must have the same or higher dye content per deposit than controls).
2. The internal capacity of the genotypes is comparable, or it is different in the same direction as the difference in defecation rate (e.g. a genotype with higher defecation rate than controls must have the same or higher internal capacity than controls). This controls for differences in food retention.
3. Flies have been pre-exposed to labelled food for at least 24 hours to ensure that they have reached steady-state conditions as regards dye accumulation.

Supplemental Experimental Procedures

Fly stocks and husbandry

The following fly stocks were used: *foxo*^{21} (Junger et al., 2003), *foxo*$^{Df(3R)Exel8159}$ (Parks et al., 2004), *ovo*^{D1} (Busson et al., 1983), *SP*^{0}, *SP*$^{\Delta130}$ and *SP*$^{+}$ control (Liu and Kubli, 2003), *chic*^{01320} (Cooley et al., 1992), *egl*^{PR29} and *egl*^{WU50} (Schupbach and Wieschaus, 1991), *Tbh*^{nM18} (Monastirioti, 2003), *elav-Gal4* (Lin and Goodman, 1994), *P0163-Gal4* (Hummel et al., 2000), *ppk-Gal4* (Ainsley et al., 2003), *HGN1-Gal4* (corresponding to the D insertion of *RN2-Gal4,* (Fujioka et al., 2003), *Ilp7-Gal4* (Yang et al., 2008), *Ilp2-Gal4* (Ikeya et al., 2002), *LK-Gal4* (de Haro et al., 2010), *tubulin-Gal80ts10* (McGuire et al., 2003), *n-syb-Gal4* (Krashes et al., 2009), *OK371-Gal4* (Mahr and Aberle, 2006), *cha7.4-Gal4* (Kitamoto, 2001), *odd-Gal4* (Larsen et al., 2006), *tubulin-Gal4* (Lee and Luo, 1999), *UAS-CD8-GFP* (Lee and Luo, 1999), *UAS-myr-mRFP* (hop B2, following re-mobilization of the original hop contributed to the Bloomington Stock Center by H. Chang), *UAS-Dscam17.1-GFP* (Wang et al., 2004), *UAS-kir2.1* (Baines et al., 2001) and *UAS-dTrpA1* (Hamada et al., 2008), UAS-*Dcr2* (Dietzl et al., 2007), *UAS-SPR-RNAi* (Yapici et al., 2008), *UAS-LKR-RNAi* and *UAS-LK-RNAi* (Dietzl et al., 2007). *Oregon R* (*OreR*) and *w*^{1118} were used as control flies.

All crosses and experiments were kept at 25ºC except for the short-term starvation experiment, which was kept at room temperature to facilitate analysis. Four- to seven-day old flies were used for all experiments. For adult-specific neuronal manipulation involving the use of *dTrpA1* or *Gal80*^{ts}, specimens were raised at 18-22°C until adulthood and were switched to 29°C on day 13-15 after eclosion. Males were used for all experiments unless otherwise indicated in order to avoid phenotypes secondary to egg laying and/or production. Flies were raised using a standard cornmeal/agar diet (1.2% autolysed yeast, 5.5% cornmeal, 6% dextrose, 0.55% agar supplemented with 0.18% Nipagin and 2.9ml/l Propionic acid) except for dietary restriction experiments where a more defined diet was required (see main text). Flies were not anesthetized prior to their transfer to experimental vials. All experiments (except for the 11-hour starvation, Figure 7A) were conducted over 24-hour periods or multiples thereof to control for circadian effects.

Immunohistochemistry

Adult tissues were dissected and fixed in 4% paraformaldehyde for 20 minutes. Subsequent washes and incubations were done in PBS with 0.2% Triton. Tissues were incubated overnight with primary antibody at 4°C, followed by a two-hour incubation with secondary antibodies at room temperature the next day. Antibodies used were: rabbit α-LK (Chen et al., 1994) (1:1,000), rabbit α-Ilp7 (Miguel-Aliaga et al., 2008) (1:5,000), goat α-green fluorescent protein (GFP) (Abcam, 1:2,000), mouse α-nc82 (Developmental Studies Hybridoma Bank, 1:50) and mouse α-elav (Developmental Studies Hybridoma

Bank, 1:75). Phalloidin-Cy5 (Molecular Probes) was used at 1:200. FITC-, Cy3-, and Cy5-conjugated secondary antibodies were obtained from Jackson Immunolabs and used at 1:200 (1:100 for the Cy5-conjugated antibody). Images were acquired using a Leica SP5 confocal microscope.

Statistical analyses

All estimations of average, shown ± standard error of the mean, were tested for significance using Mann-Whitney's *U* test unless a normality test (Shapiro-Wilk normality test) was passed with $p > 0.05$, in which case a Student's *t*-test was used. The significance of repeated experiments was calculated using Fisher's combined probability test; in such cases, the figure shows a representative experiment and the combined p-value is given in the legend. Distributions (such as hue distributions) were compared using a two-sample Kolmogorov-Smirnov test. Boxplot graphs display 10th and 90th percentile as whiskers, 25th and 75th as box extremes, and the median as the box band. Linear regression was performed by fitting a linear model with the least squares method and applying F-statistic to obtain a p-value. All statistical analyses were performed using the R environment (R Development Core Team, 2009).

Full genotypes used in Figures 5-7

Figures 5E-5F: w^{1118}*; HGN1-Gal4/+; +; + (Gal4* control); w^{1118}*; UAS-kir2.1/HGN1-Gal4; +; + (HGN1>kir2.1)*; w^{1118}*; UAS-kir2.1/+; +; +* (*UAS* control).

Figure 6C: w^{1118}*; LK-Gal4/+; +; + (Gal4* control); w^{1118}*; UAS-dTrpA1/LK-Gal4; +; + (LK>dTrpA1)*; w^{1118}*; UAS-dTrpA1/+; +; +* (*UAS* control). Figures 6E and 6H-6J: w^{1118}*; tubulin-*$Gal80^{ts}$*, LK-Gal4/+; +; + (Gal4* control); w^{1118}*; tubulin-*$Gal80^{ts}$*, LK-Gal4/UAS-kir2.1; +; + (LK>kir2.1)*; w^{1118}*; UAS-kir2.1/+; +; + (UAS* control).

Figures 7F and 7G: w^{1118}*; +; Ilp2-Gal4/+*; + *(Gal4* control); w^{1118}*; UAS-kir2.1/+; Ilp2-Gal4/+; + (Ilp2>kir2.1)*; w^{1118}*; UAS-kir2.1/+; +; +* (*UAS* control). Figures 7H and 7I: w^{1118}*; +; Ilp7-Gal4/+; + (Gal4* control); w^{1118}*; UAS-kir2.1/+; Ilp7-Gal4/+; + (Ilp7>kir2.1)*; w^{1118}*; UAS-kir2.1/+; +; +* (*UAS* control).

Supplemental References

Ainsley, J.A., Pettus, J.M., Bosenko, D., Gerstein, C.E., Zinkevich, N., Anderson, M.G., Adams, C.M., Welsh, M.J., and Johnson, W.A. (2003). Enhanced locomotion caused by loss of the Drosophila DEG/ENaC protein Pickpocket1. Curr Biol *13*, 1557-1563.

Baines, R.A., Uhler, J.P., Thompson, A., Sweeney, S.T., and Bate, M. (2001). Altered electrical properties in Drosophila neurons developing without synaptic transmission. J Neurosci *21*, 1523-1531.

Busson, D., Gans, M., Komitopoulou, K., and Masson, M. (1983). Genetic Analysis of Three Dominant Female-Sterile Mutations Located on the X Chromosome of DROSOPHILA MELANOGASTER. Genetics *105*, 309-325.

Chen, Y., Veenstra, J.A., Davis, N.T., and Hagedorn, H.H. (1994). A comparative study of leucokinin-immunoreactive neurons in insects. Cell Tissue Res *276*, 69-83.

Cooley, L., Verheyen, E., and Ayers, K. (1992). chickadee encodes a profilin required for intercellular cytoplasm transport during Drosophila oogenesis. Cell *69*, 173-184.

de Haro, M., Al-Ramahi, I., Benito-Sipos, J., Lopez-Arias, B., Dorado, B., Veenstra, J.A., and Herrero, P. (2010). Detailed analysis of leucokinin-expressing neurons and their candidate functions in the Drosophila nervous system. Cell Tissue Res *339*, 321-336.

Dietzl, G., Chen, D., Schnorrer, F., Su, K.C., Barinova, Y., Fellner, M., Gasser, B., Kinsey, K., Oppel, S., Scheiblauer, S., et al. (2007). A genome-wide transgenic RNAi library for conditional gene inactivation in Drosophila. Nature *448*, 151-156.

Fujioka, M., Lear, B.C., Landgraf, M., Yusibova, G.L., Zhou, J., Riley, K.M., Patel, N.H., and Jaynes, J.B. (2003). Even-skipped, acting as a repressor, regulates axonal projections in Drosophila. Development *130*, 5385-5400.

Hamada, F.N., Rosenzweig, M., Kang, K., Pulver, S.R., Ghezzi, A., Jegla, T.J., and Garrity, P.A. (2008). An internal thermal sensor controlling temperature preference in Drosophila. Nature *454*, 217-220.

Hummel, T., Krukkert, K., Roos, J., Davis, G., and Klambt, C. (2000). Drosophila Futsch/22C10 is a MAP1B-like protein required for dendritic and axonal development. Neuron *26*, 357-370.

Ikeya, T., Galic, M., Belawat, P., Nairz, K., and Hafen, E. (2002). Nutrient-dependent expression of insulin-like peptides from neuroendocrine cells in the CNS contributes to growth regulation in Drosophila. Curr Biol *12*, 1293-1300.

Junger, M.A., Rintelen, F., Stocker, H., Wasserman, J.D., Vegh, M., Radimerski, T., Greenberg, M.E., and Hafen, E. (2003). The Drosophila forkhead transcription factor FOXO mediates the reduction in cell number associated with reduced insulin signaling. J Biol *2*, 20.

Kitamoto, T. (2001). Conditional modification of behavior in Drosophila by targeted expression of a temperature-sensitive shibire allele in defined neurons. J Neurobiol *47*, 81-92.

Krashes, M.J., DasGupta, S., Vreede, A., White, B., Armstrong, J.D., and Waddell, S. (2009). A neural circuit mechanism integrating motivational state with memory expression in Drosophila. Cell *139*, 416-427.

Larsen, C., Franch-Marro, X., Hartenstein, V., Alexandre, C., and Vincent, J.P. (2006). An efficient promoter trap for detection of patterned gene expression and subsequent functional analysis in Drosophila. Proc Natl Acad Sci U S A *103*, 17813-17817.

Lee, T., and Luo, L. (1999). Mosaic analysis with a repressible cell marker for studies of gene function in neuronal morphogenesis. Neuron *22*, 451-461.

Lin, D.M., and Goodman, C.S. (1994). Ectopic and increased expression of Fasciclin II alters motoneuron growth cone guidance. Neuron *13*, 507-523.

Liu, H., and Kubli, E. (2003). Sex-peptide is the molecular basis of the sperm effect in Drosophila melanogaster. Proc Natl Acad Sci U S A *100*, 9929-9933.

Mahr, A., and Aberle, H. (2006). The expression pattern of the Drosophila vesicular glutamate transporter: a marker protein for motoneurons and glutamatergic centers in the brain. Gene Expr Patterns *6*, 299-309.

McGuire, S.E., Le, P.T., Osborn, A.J., Matsumoto, K., and Davis, R.L. (2003). Spatiotemporal rescue of memory dysfunction in Drosophila. Science *302*, 1765-1768.

Miguel-Aliaga, I., Thor, S., and Gould, A.P. (2008). Postmitotic specification of Drosophila insulinergic neurons from pioneer neurons. PLoS Biol *6*, e58.

Monastirioti, M. (2003). Distinct octopamine cell population residing in the CNS abdominal ganglion controls ovulation in Drosophila melanogaster. Dev Biol *264*, 38-49.

Parks, A.L., Cook, K.R., Belvin, M., Dompe, N.A., Fawcett, R., Huppert, K., Tan, L.R., Winter, C.G., Bogart, K.P., Deal, J.E., et al. (2004). Systematic generation of high-resolution deletion coverage of the Drosophila melanogaster genome. Nat Genet *36*, 288-292.

Perrimon, N., and Gans, M. (1983). Clonal analysis of the tissue specificity of recessive female-sterile mutations of Drosophila melanogaster using a dominant female-sterile mutation Fs(1)K1237. Dev Biol *100*, 365-373.

R Development Core Team. (2009). R: A Language and Environment for Statistical Computing.

Schupbach, T., and Wieschaus, E. (1991). Female sterile mutations on the second chromosome of Drosophila melanogaster. II. Mutations blocking oogenesis or altering egg morphology. Genetics *129*, 1119-1136.

Verheyen, E.M., and Cooley, L. (1994). Profilin mutations disrupt multiple actin-dependent processes during Drosophila development. Development *120*, 717-728.

Wang, J., Ma, X., Yang, J.S., Zheng, X., Zugates, C.T., Lee, C.H., and Lee, T. (2004). Transmembrane/juxtamembrane domain-dependent Dscam distribution and function during mushroom body neuronal morphogenesis. Neuron *43*, 663-672.

Yang, C.H., Belawat, P., Hafen, E., Jan, L.Y., and Jan, Y.N. (2008). Drosophila egg-laying site selection as a system to study simple decision-making processes. Science *319*, 1679-1683.

Yapici, N., Kim, Y.J., Ribeiro, C., and Dickson, B.J. (2008). A receptor that mediates the post-mating switch in Drosophila reproductive behaviour. Nature *451*, 33-37.
