# Supplementary figures and images for: Enteric Neurons and Systemic Signals Couple Nutritional and Reproductive Status with Intestinal Homeostasis
Source: Cell Metab. 2011 Jan 5;13(1):92–104. doi: 10.1016/j.cmet.2010.12.010 (PMC3038267; doi:10.1016/j.cmet.2010.12.010)

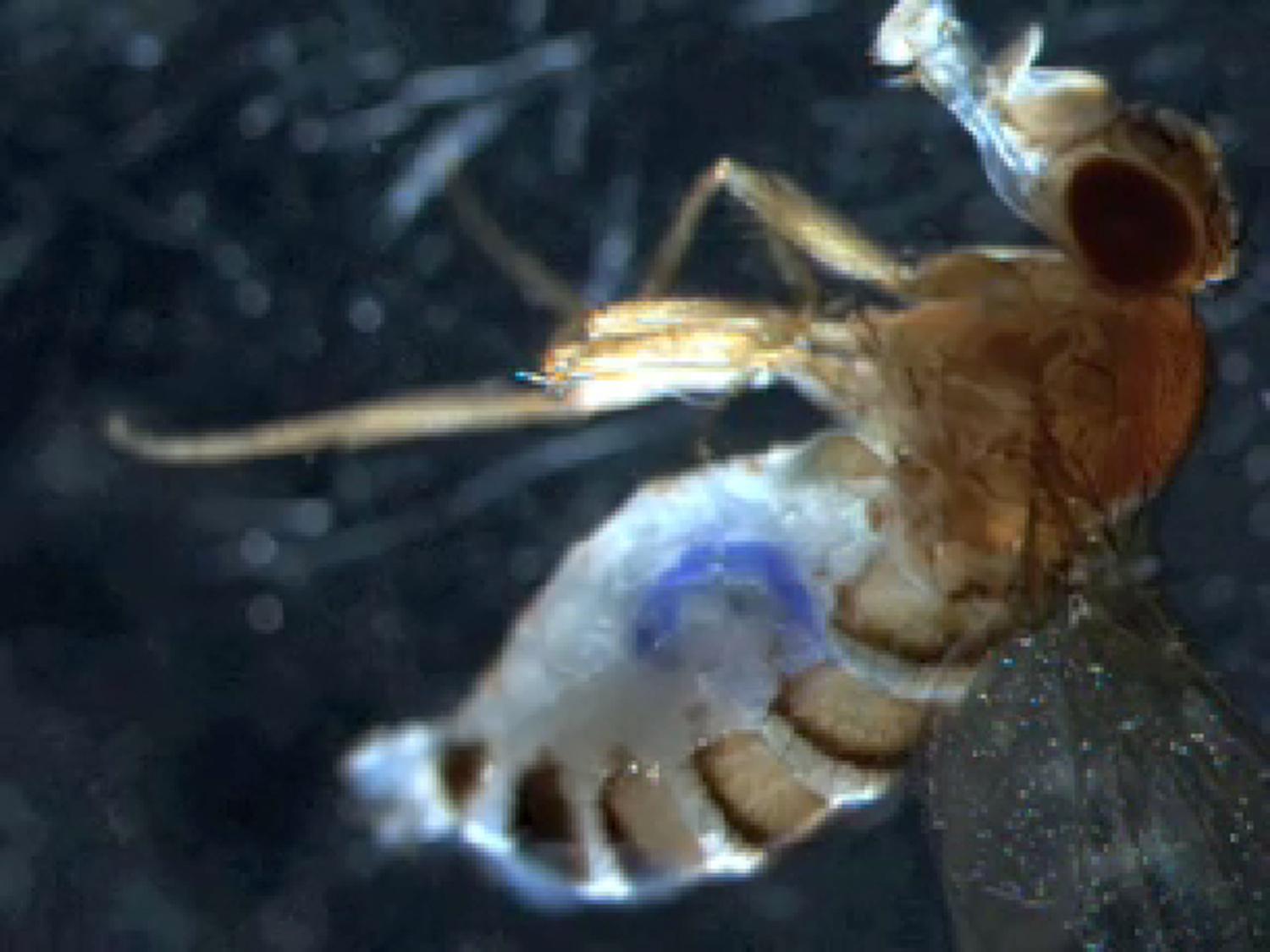

Supplement: Movie S1. Increased Fluid Retention upon Genetic Inactivation of LK Neurons — Related to Figure 6. Insertion of the end of a pair of forceps in the abdomen of a bloated LK-silenced fly in PBS reveals that swelling is caused by fluid retention; note the resulting deflation and the absence of air bubbles. [file mmc2.jpg]
